# Balancing sufficiency and impact in reporting standards for mass spectrometry imaging experiments

**DOI:** 10.1093/gigascience/giy102

**Published:** 2018-08-14

**Authors:** Ove J R Gustafsson, Lyron J Winderbaum, Mark R Condina, Berin A Boughton, Brett R Hamilton, Eivind A B Undheim, Michael Becker, Peter Hoffmann

**Affiliations:** 1 ARC Centre of Excellence in Convergent Bio-Nano Science & Technology (CBNS), University of South Australia, Mawson Lakes, South Australia 5095, Australia; 2 Future Industries Institute, University of South Australia, Mawson Lakes, South Australia 5095, Australia; 3 Metabolomics Australia, School of BioSciences, University of Melbourne, Parkville, Victoria 3010, Australia; 4 Centre for Microscopy and Microanalysis, University of Queensland, St. Lucia, Queensland 4072, Australia; 5 Centre for Advanced Imaging, University of Queensland, St. Lucia, Queensland 4072, Australia; 6 Boehringer Ingelheim Pharma GmbH & Co. KG, Biberach a.d. Riss 88397, Germany

**Keywords:** mass spectrometry, imaging, reporting, standard

## Abstract

Reproducibility, or a lack thereof, is an increasingly important topic across many research fields. A key aspect of reproducibility is accurate reporting of both experiments and the resulting data. Herein, we propose a reporting guideline for mass spectrometry imaging (MSI). Previous standards have laid out guidelines sufficient to guarantee a certain quality of reporting; however, they set a high bar and as a consequence can be exhaustive and broad, thus limiting uptake.

To help address this lack of uptake, we propose a reporting supplement—Minimum Information About a Mass Spectrometry Imaging Experiment (MIAMSIE)—and its abbreviated reporting standard version, MSIcheck. MIAMSIE is intended to improve author-driven reporting. It is intentionally not exhaustive, but is rather designed for extensibility and could therefore eventually become analogous to existing standards that aim to guarantee reporting quality. Conversely, its abbreviated form MSIcheck is intended as a diagnostic tool focused on key aspects in MSI reporting.

We discuss how existing standards influenced MIAMSIE/MSIcheck and how these new approaches could positively impact reporting quality, followed by test implementation of both standards to demonstrate their use. For MIAMSIE, we report on author reviews of four articles and a dataset. For MSIcheck, we show a snapshot review of a one-month subset of the MSI literature that indicated issues with data provision and the reporting of both data analysis steps and calibration settings for MS systems. Although our contribution is MSI specific, we believe the underlying approach could be considered as a general strategy for improving scientific reporting.

## Background

Our specific research field—mass spectrometry imaging (MSI) [1]—applies mass spectrometry (MS) for the raster-based collection of mass spectra from a discrete set of locations on the surface of a two-dimensional sample. This can be achieved for synthetic (human-made materials) and natural (geological) surfaces or, as is more common, biological cross-sections of plant and animal tissues. Depending on sample preservation, preparation, and MS settings, MSI can investigate the spatial distributions for a wide variety of analytes, including small molecules (drugs, lipids) [2], *N*-glycans [3] or peptides, and proteins [4]. MSI measures the molecular composition of a sample, simultaneously acquiring information on hundreds to thousands of compounds, in some cases, across multiple compound classes, without prerequisite knowledge of composition and without compound-specific labeling (e.g., antibodies) [5]. The spatial component of the information obtained by MSI is orthogonal to most omics approaches, which generally do not consider the spatial aspect of global abundance changes [6].

It is in the MSI context that we address the current lack of standardization in reported research. MSI is arguably one of the most challenging experimental approaches in MS and, similar to other MS-based technologies, is data intensive. Combined with the numerous approaches available for sample preparation and analysis, the care required during sample preparation [7], as well as the observation that dedicated training is necessary to master it [8], there is a need for highly detailed reporting. However, adherence to the available reporting standards is not the norm, and MSI requires a substantial step toward standardized acceptable levels of reporting. This is important for building confidence in the reproducibility of experimental results, both for method development and in primary research. The importance of this issue is exemplified by the so-called reproducibility crisis, which is currently being widely discussed in the wider literature and is not MSI specific [9]. One of the core concepts affecting reproducibility is insufficient quality checks and reporting at both the experimental and data processing levels [10]. These are the gaps that we propose to address.

### Reproducibility and the current reporting standards

Publication remains *the* primary method for result dissemination in the sciences. Transparency, accuracy, and completeness in these reports form a cornerstone of reproducibility. Unfortunately, even though the “crisis” is topical, discussions on addressing it are hampered by the fact that reproducibility is typically not well defined [11]. We propose to adopt the three-part lexicon of Goodman et al. [11] in order to help clarify this ambiguity. These three parts are methods, results, and inferential reproducibility. We focus on the first two of these in our discussion. Briefly, methods reproducibility is the ability to reproduce the exact data analysis and arrive at identical end results. For example, a publication would be methods reproducible if, using the same analysis methods on the same raw data, one could arrive at the same outputs (i.e., figures) as those reported in that publication. There is a degree of subjectivity in the choice of what results are presented in a figure. However, once that decision has been made, the reproduction of that figure from the raw data should be completely deterministic, and all the details required for reproduction should be included in the publication/report. In contrast, results reproducibility is the ability of other groups to follow the reported procedures *as closely as practical*, generate new data, and arrive at similar results. This requires that sufficient detail be reported about these procedures and spans sample preparation, data acquisition, the data itself, as well as its processing and analysis [11].

To achieve methods and results reproducibility [11], reporting in publications must obviously meet a certain quality standard. One might expect a minimum quality based on the scientific method—scientists are driven to methodically replicate, validate, and report their findings—that, combined with journal guidelines [12] and the availability of alternate publication formats such as protocols (*Rapid Communications in Mass Spectrometry*), data briefs (*Data-in-Brief*), and video journals (*Journal of Visualised Experiments*), would suggest that all the pieces needed for high-quality reporting are in place. Despite this resource-rich environment, there are remaining issues in reporting. Existing standards such as the Minimum Information About a Proteomics Experiment (MIAPE) [13] guidelines were added in an effort to prompt tangible improvements in reporting: to set the bar by defining peer expectations of reporting and therefore, hopefully, have a tangible impact on reproducibility. There are several other existing standards that are also MSI relevant and aim to encompass different research communities. One of the broadest is the minimum information for biological and biomedical investigations guidelines [14], which provide a framework of standards for the integrated reporting (i.e., reducing standards overlap) of biological experiments. MIAPE is a relevant standard specific to proteomics [13], and Minimum Information Required for a Glycomics Experiment (MIRAGE) is similar but designed for glycomics [15]. MIAPE is compartmentalized further to include, e.g., MIAPE-MS [16], which is the most MSI-relevant component of MIAPE. MIRAGE has also expanded to encompass sample preparation [17], MS analysis [15], and glycan microarray analysis [18]. These standards specify a minimum level of reporting quality by identifying a set of information that should be included when reporting an experiment and that is sufficient to guarantee that the report will meet that specified level of quality.

One way to determine if research is being reported well would be to evaluate its methods reproducibility. Obviously, evaluating results reproducibility is the ideal, but it is expensive and difficult to justify replication studies [19, 20]. Conversely, confirming methods reproducibility is not easy in practice but ultimately *should*at least be possible, if not also straightforward. While such confirmation does not directly evaluate research quality, it does evaluate reporting quality. This is important enough that we suggest that it can be used as a cost-effective heuristic to estimate results reproducibility and hence “quality.” If we focus on methods reproducibility, it quickly becomes apparent that research is not being reported well. In effect, an individual could not repeat the study without further input from the authors [20]. This is despite the existence of well-defined standards and is a key component of the reproducibility crisis. Crucially, this is not a reflection on the quality of existing reporting standards, as they highlight core methods that require reporting and raise valid discussion points. However, they are either being misinterpreted—the assumption being that all scientists will interpret a reporting requirement similarly—or simply not being used. We suggest that an underlying cause of this is that standards typically aim to set a bar for reporting quality far above the current norm: they aim to be both necessary and sufficient. In practice they end up being sufficient, but not necessary, conditions for reporting quality.

### Sufficiency vs necessity: sacrificing sufficiency for practicality

To illustrate the concepts of necessary and sufficient conditions for reporting quality, we describe the two fundamental approaches one could take to construct a standard that is both sufficient and necessary. One approach would be to start with a list of experimental details that, together, are sufficient but not necessary. As many details as possible would then be removed while maintaining sufficiency. Eventually, if the details are granular enough, the last superfluous detail would be removed, and no other details could be removed while still maintaining sufficiency. At this point, the standard would not only be sufficient but also necessary to achieve the specified level of quality. This is the approach the existing standards take. Conversely, one could start from a blank slate—no experimental details—and add a necessary detail, such as provision of raw data. This would now be necessary, but not sufficient, for achieving the given level of reporting quality. One could then continue to add necessary experimental details until sufficiency is reached. This is the approach we use with Minimum Information About a Mass Spectrometry Imaging Experiment (MIAMSIE). We are not setting a fixed bar, but introducing a bar that can be steadily lifted as the field adopts it. Note that although having a standard that is both sufficient and necessary would be the best-case scenario, we are not claiming that this is realistic. Rather, we claim that the approach of starting with a sufficient standard and iterating from there has not produced the intended improvements in the field. Taking a different approach, such as starting from a necessary standard and iterating in the other direction, could potentially produce more tangible improvements in reporting quality as it would produce intermediate steps that are easier to implement.

The requirement for sufficiency in existing standards necessitates broad, all-encompassing, and consequently ambiguous phrasing. This can result in the unintentional omission of experimental detail(s). For example, in MIAPE-MS 2.98 [21], the *“Acquisition parameters”* are required. The problem with broadly phrased detail requests such as these is that they allow for equally broad responses, such as *“the default acquisition parameters were used.”* Here, an assumption might be that reporting default settings for a company-installed and therefore “standard” system is sufficient to encompass *“Acquisition parameters.”* Another example from MIAMPE-MS 2.98 is that *“Parameters used in the generation of peak lists or processed spectra”* are required. In this example, if peak lists were generated by smoothing, baseline reduction, and peak picking, a description of only the smoothing and peak-picking parameters would still technically satisfy *“Parameters used in the generation of peak lists.”* Unfortunately, such inconsistencies are difficult to audit. The only way to conclusively determine that the methods described are insufficient to reproduce an analysis would be to apply the methods to the raw data and demonstrate different results. This is not standard practice during peer review and, as we will demonstrate, is not realistic given the lack of raw data provision in MSI. One goal of reporting should be to convince reviewers and the community that reproduction would be possible given time and resources, not to complicate design or data such that reproduction is deemed impractical.

We stress at this point that our assumption is not that omission is intentional but that it results from the lack of clarity in reporting standards, the lack of uptake, the complexity of contemporary experimental procedures, the “assumed knowledge” afforded to some process steps, and the time pressure for publication. In the absence of automated data and meta-data harvesting [22], a human-driven reporting standard could compensate for human error by directly prompting the inclusion of as many necessary experimental details as possible [15]. Furthermore, such granular standards that directly prompt inclusion of very specific information also allow for auditing of existing reports with relative ease in comparison to the existing broad standards for which this would be relatively difficult. Easy auditing could be of interest to authors, reviewers, journals, or any other individual or organization for whom systematic reporting quality control is key. Standards that allow auditing with relative ease could also be translated to in-house record-keeping processes, thereby not only reducing the time required for translation of research notes to publication format but also making inter-laboratory comparisons, quality control, and standardization consistent and practical.

### A two-part approach to granular MSI reporting standards

Misinterpretation of standards due to broad and vague phrasing can be minimized by creating more granular standards, in which the required information is defined more explicitly [23]. As a side effect, an increase in specificity may also lead to greater ease of use and therefore greater reporting compliance [24]. However, granular and explicit terms make it unrealistic to be broad and all-encompassing, at least initially before such standards have had time to evolve as the research community interacts with them. Because of this, any initial proposed standard will preferentially favor a certain subset of methods and experimental designs. It should be noted that we, and others before us, do not intend for granularity to subsequently imply the preferred use of particular methods or experimental designs; we aim to address structured reporting only [15, 25]. We also claim that progressive standards iteration and collaboration should produce improvements that will incrementally remove any such researcher-driven bias, as the standards gradually evolve to become more broad and all-encompassing while also reflecting the true method and design biases present in the MSI field.

Granular items of necessary detail are exemplified by the recent suggestion to include negative controls for analyte delocalization in MSI [26]. This would involve the inclusion of off-tissue spectra in every MSI data acquisition region. The suggestion is a good one and necessary to evaluate sample preparation quality. However, it is narrow in scope and, alone, it is far from sufficiency. At the other end of the spectrum, the MIRAGE guidelines are a good example of a complete granular standard—to the point of having an explicit list of fields to be filled in. This typifies the prompting concept and is a consequence of the specific requirements of glycomics, as related to the complexity of glycans and their fragility during MS analysis [15].

A granular standard was proposed for MSI in 2012 on the Mass Spectrometry Imaging Society (MSIS) forum [27]. Unfortunately, the MSI community did not seem to engage with this initiative. The last contribution was in June 2012 and is currently not accessible. McDonnell et al. continued the discussion in 2015 by suggesting a working document for an updated granular MSI extension of MIAPE [23]. The suggested standard focused on the “minimum information that is necessary to adequately describe an MSI experiment”—i.e., it aims to be both necessary and sufficient [23]. Additional recommendations, as also suggested by sources that include MIAPE and MIRAGE, were that a standard should be able to grow with the field (i.e., be stable and modular)—and balance completeness of reporting (adding reporting details) with practicality, or operational uptake [13, 15, 23]. Although potentially less powerful, a practical and, therefore, minimalist standard makes implementation easier. This approach may be *the* driver needed for tangible reporting improvements. Since then, there has been a need to continue the reporting standards discussion initiated by the MSIS and prompt the community to engage effectively with a standard.

In this context, we propose a template of reporting fields, MIAMSIE, that directly prompts the collection of very specific granular information, reminiscent of a methods section that lists materials and processes, rather than providing this as prose. This allows not only straightforward completion but also rapid quantitative evaluation of adherence to the standard when collected in a standardized format. Collating this information is a Herculean task for published studies that follow a sufficient guideline but provide this information as unstructured prose.

Given that current sufficient standards suffer from low uptake rates, we also propose that MIAMSIE should initially be abbreviated to prioritize uptake over completeness: a brevity-with-impact concept. The abbreviated reporting template was named MSIcheck. As is to be expected, both the full and abbreviated versions are suited for different tasks. MIAMSIE is an author-driven reporting aid intended to prompt the user into providing a number of key pieces of granular information [15], represented as a list of fields that are important for reporting an MSI experiment. MIAMSIE is designed for expansion, to adapt to user needs so as to gradually become more comprehensive over time. To demonstrate the concept proposed, we have focused on laser desorption/ionization (LDI)-specific MSI. However, the intent is for community engagement to drive expansion to encompass the entirety of the field. The key is to ensure that the ultimate aim of sufficiency in a mature MSI standard is not lost. In effect, the current version of MIAMSIE does not aim to guarantee reporting quality but is intended as a step toward this guarantee. MSIcheck makes implementation as easy as possible. It is important to note that it is not a separate standard but rather a subset of MIAMSIE that is designed to be very easy to use in order to achieve more rapid impact. Instead of setting the bar for reporting quality, MSIcheck focuses on absolute key aspects of a report in order to evaluate the current state of the field and identify problem points that need to be prioritized. As we outline herein, the aim is for the fields in MSIcheck to grow along with uptake, thereby initially prioritizing uptake over impact, with the ultimate end goal being a MIAMSIE standard that encompasses the entire MSI field.

Here, we discuss the genesis, structure, and scope of MIAMSIE and the abbreviated reporting template MSIcheck, the improvement we anticipate will be brought to the field, and the results of implementing both to reporting of publications and datasets (MIAMSIE) and for review of the MSI literature (MSIcheck).

## MIAMSIE

### The purpose and scope of MIAMSIE

MIAMSIE (Supplementary Table S1) is a conglomerate list of experimental reporting fields that directly draws upon and consolidates several existing resources in MS. The core standard is based on the MIAPE imaging export template provided in the R package Cardinal [28, 29], as well as consideration of the reporting requirements of MIAPE [13] and specifically the MIAPE-MS module [16], the MSI data standard imzML [30], and the MSI standard proposed by McDonnell et al. in 2015 [23]. In addition, field names and descriptions are either verbatim, as in contributing standards such as MIAPE imaging, or modified based on the requirements of the conglomerate MIAMSIE standard.

Note that the intent of MIAMSIE is not to provide a complete, field-wide, reporting template and mechanism that will gold-plate adherence to a standard. If the concept is popular, MIAMSIE should evolve to encompass existing ontologies, the requirements of varied experimental MSI niches, and a tool that streamlines provision of these reports in a structured format that can also be examined effectively by nonexperts. This is beyond the scope of the discussion presented here but is the ultimate driver of this work.

The intended use of MIAMSIE is as a reporting supplement for:

- Primary research articles, protocols, and data briefs;
- Registered reports describing an experiment [10], e.g., accompanying a submission to a data repository (PRoteomics IDEntifications [PRIDE] [31]);
- Teaching of structured reporting (in-house or at workshops/conferences); and
- Regular day-to-day in-house record-keeping.

MIAMSIE currently focuses heavily on laser LDI-MSI and, in particular, matrix-assisted laser desorption/ionization (MALDI)-MSI. This is deliberate, as this is our main technology platform, but also as we intend to demonstrate how such a standard could be used to improve reporting. Obviously, this means the inclusion of many MALDI-specific fields. The intention is that users will add fields as they need them (e.g., secondary ion mass spectrometry (SIMS)-specific fields), gradually making MIAMSIE more comprehensive. A core philosophy of MIAMSIE is to be as unambiguous as practical with regards to the type and amount of information requested. As a result, the individual entries are very granular. This granularity, combined with the focused scope of the initial standard, mean that the standard does not currently guarantee a sufficient quality level for reporting across all MSI studies. This will only be possible if the standard is used and expanded, thereby gradually raising the bar. Assuming high uptake, it is hoped that MIAMSIE, or a potential successor, will eventually be able to guarantee a sufficient level of reporting quality for MSI, in much the same way that existing standards such as MIAPE intend. MIAMSIE aims to provide standardized reporting and also encourage further discussion and development (i.e., tools for reporting).

### Terminology used in the MIAMSIE standard

The terminology used in MIAMSIE is intended to minimize ambiguity; however, there are key terms that are left necessarily broad due to the variety of experiments that MIAMSIE is intended to capture. These include *experiment*, *sample*, and *dataset*. An experiment includes experimental design, hypotheses and aims, the entire collection of samples used, the sample preparation, the datasets that have been collected, as well as the data analysis used. Essentially, the term *experiment* is used to describe the entire study that MIAMSIE is being used to document and is often directly related to a publication. A *sample* refers to the surface to which MSI is applied and can be from a synthetic, natural, or biological source. Often an *experiment* will collect data from several *samples* with different sources. These *samples* may differ either internally or from each other or may originate from different categories (biological/clinical) that are directly relevant to the hypotheses. A *dataset* refers to a single acquisition area or region on a *sample* from which a collection of spectra have been acquired. Typically, these spectra will have been collected using virtually identical acquisition parameters across all *samples*.

The reason it is important to leave *sample* and *dataset* relatively broad is that there exist MIAMSIE fields that will require different values for different experiment types. As a result, the terms *sample* and *dataset* need to label these experiments in a way that is useful. The most reasonable way to report these different values will naturally differ across experiment types. For example, in an experiment where a murine kidney is sectioned and analyzed by MSI and a subset of sections are treated with an enzyme to release *N*-glycans [32], *samples* could be defined as “enzyme treated” or “control.” This allows for the sample preparation steps to be assigned either of these two values for each sample, with each identified by the appropriate sample name, thereby unambiguously defining which sections of tissue were treated with what preparation procedure while still being as concise as possible. To use an additional example, if the laser power or some other data acquisition parameter was optimized for each section, then the sections could be defined as separate datasets, each with a unique identifying dataset name, and the laser power for each one would be identified using these names. Datasets would then be linked to samples, i.e., which datasets are in which samples and *vice versa*. Essentially, the intention is for samples and datasets to provide useful partitions of the data into subsets that differ in sample preparation, origin, or data acquisition parameters. The exact way in which the data are partitioned will differ in any given experiment, and so the user should be mindful of defining these terms in the context of the experiment clearly and carefully and should be consistent with their definitions. As an additional example, these definitions can be included as part of the *Value(s)* for the *Sample* and *Dataset* fields or be defined in the publication with appropriate reference to the MIAMSIE field in question.

### The structure of MIAMSIE

The initial premise was that to satisfy MIAMSIE, a value needed to be provided for every field. However, during development, it became clear that a large number of fields are only required conditionally. In effect, they depend on the answer to a “gating question” such as “Was LDI used for ionization?” To continue with this example, if the answer is “Yes,” values for all the LDI-dependent fields need to be provided, and if the answer is “No,” these fields are not applicable. To help navigate MIAMSIE, conditional fields were grouped as much as possible and the list annotated with the relevant gating questions.

Each individual field in the template (Supplementary Table S1) has an *ID* (a unique identifier), a *Name* (a short name for the field), a *Description* (a more detailed explanation of the information requested by the field), a *Category* (useful for organizing information), a list of *Valid Values* for the field, and an empty column where a *Value* could be provided for a given field and experiment. These valid values improve MIAMSIE's usability by providing guidance on completion of the required fields. In the *Description* for a field, it may reference another field in the format *#(AAA)* where *#* is the *ID* for the field being referenced and *AAA* is its *Name*. If a field is not relevant to a particular experiment, a value of *NA* is accepted and is indicated at the beginning of the *Valid Values* characteristic for that field. This identifies the conditional fields mentioned above, and the purpose of the gating questions is essentially to identify the fields that can be ignored (given a value of *NA*). Some fields only have a limited number of possible values, in which case they are listed in the *Valid Values* characteristic for that field. Other fields have valid values not listed in *Valid Values*, and these are identified by the entry ending with an ellipsis (…).

**Figure 1:**
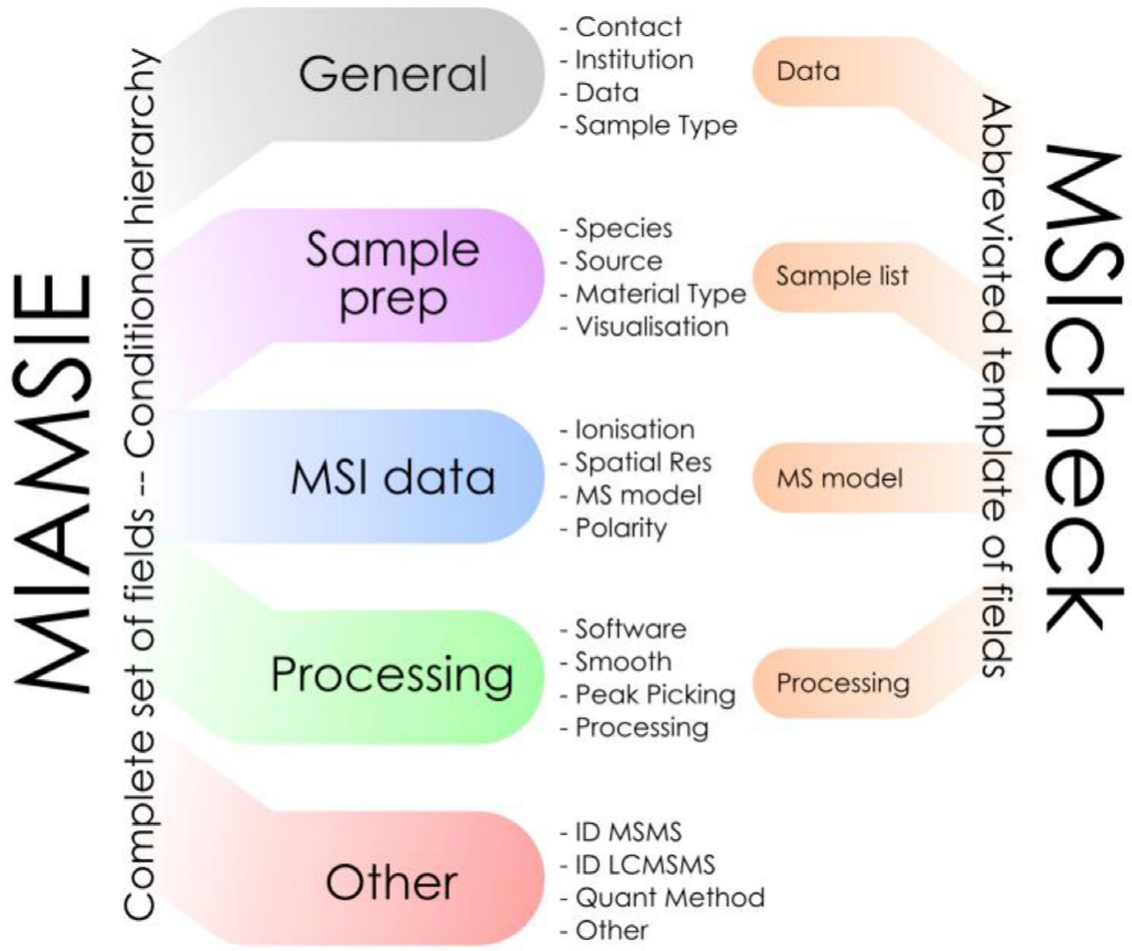
Schematic overview of categories and example fields from MIAMSIE and how the complete list of fields was abbreviated to form the more succinct MSIcheck standard.

Each field in MIAMSIE belongs to one of five possible categories (see Fig. 1): *General, Sample Preparation, MSI Data, Processing*, and *Other*. Of these categories, *General* contains fields that provide links to additional information, experiment-wide details (*Aim, Hypothesis*), identifying names for datasets and samples (*Dataset Names* & *Sample List*), and fields providing information about how datasets and samples are related (*Sample Disambiguation*). Fields in the *General* category are broadly applicable and could therefore serve as a starting point when adapting MIAMSIE to another scientific field. In contrast, the *Other* category provides a field for information that is specific to a particular experiment but for which a field is not provided in MIAMSIE. Examples include results validation by orthogonal MS or non-MSI methods (e.g., nuclear magnetic resonance). This provides a key functionality for MIAMSIE, allowing adopters to incrementally raise the bar for reporting quality by adding information when deemed necessary for a specific report without specifically requiring an additional mechanism (e.g., online tool or graphical user interface [GUI]). As MIAMSIE evolves and fields are added, we would expect the *Other* category to see less use, and its over-use could provide information on fields or entire MSI workflows that should be added. Intermediate categories *Sample Preparation*, *MSI Data*, and *Processing* contain many fields that could have different values for different datasets or samples and so may require the provision of multiple values. Ideally, the final iteration of this standard will use a GUI that, in addition to allowing easy recording of MIAMSIE information, will also include mechanisms for suggesting new fields. An alternative approach that could prompt open discourse regarding the content of the standard would be to host the current working MIAMSIE document on a resource such as GitHub [33], allowing monitoring, suggestions for changes, as well as forum discussions to be transparent and have greater reach. As MIAMSIE is informed by imzML, this approach could also encompass tools such as mzML2ISA, which streamlines reporting of fields that appear in mzML and imzML controlled vocabularies [22].

### MIAMSIE as a conditional hierarchy

To illustrate the way in which we anticipate that MIAMSIE will evolve with peer contribution, we briefly describe its iterative development pre-publication. Prior to its finalization, a draft list of the MIAMSIE fields was provided to our co-authors for a test application to a set of four published MSI experiments (Supplementary Tables S2 and S3) and one *in-house*dataset (Supplementary Table S4). As completed for these experiments, MIAMSIE was able to cover experimental details of interest while also allowing an overview of the study scope and processes. The template nature of the standard prompted inclusion of the requested information and was therefore straightforward to implement. However, MIAMSIE was still considered lengthy and thus potentially difficult to complete. Suggestions for improvement included clarification of the information being requested by individual fields, the addition of other MSI modalities, increasing the relevance of the provided fields to more MSI study types, and improving the ease of completion.

In line with these discussions between the authors, several changes were made to MIAMSIE while maintaining its core content, including

- The gating questions were added to create an improved template user interface. In effect, when the answer *“Yes”* is selected for a gating question, the MIAMSIE fields relevant to that question become visible, otherwise they are hidden. This ensures that, as much as practical, only relevant fields need to be considered, significantly simplifying completion of the standard and directly addressing the perceived difficulty of using MIAMSIE.
- The first additional ionization technique-specific fields were added: SIMS (e.g., *ToF Nano*).
- Fields were added to improve the relevance of the standard across a more diverse array of MSI experiments. This included extra conditionally gated fields for botanical, natural (geological), and synthetic samples, as well as making the storage and sample treatment fields more flexible. This reflected the varied analytical backgrounds of the authors.
- Fields that were important to a study but not strictly necessary for complete reporting of an experiment itself were removed (e.g., *Ethics Approval, Abstract*).
- The *Descriptions* and/or *Valid Values* for some fields were clarified, as necessary.

With these modifications, the final MIAMSIE template (Supplementary Table S1) contains a total of 130 fields. This is reduced to 81 fields with the updated user interface, assuming a single MSI acquisition with MALDI is completed for a stabilized cross-section of a biological sample that is also visualized (hematoxylin & eosin [H&E] or microscopy), with only the raw data provided (no processing or MSI visualization). As conditional answers change (e.g., ± storage or *in situ* chemistries, processing), the number of fields that need to be filled in by the user would change accordingly.

The observation that MIAMSIE needed streamlining to increase uptake was expected, but the contrast between perceived and actual difficulty of completion was stark. Although it contains more than 100 fields, one co-author estimated their completion at less than 20 minutes. This is a reasonable time frame for completion of MIAMSIE by the principal researcher(s), particularly when balanced against the amount of information being recorded. A detail-focused standard such as MIAMSIE is only feasible as a reporting scheme for authors, as they will be sufficiently familiar with their experiments to make the process practical. Nevertheless, this perception, which is likely to persist regardless, means it is unlikely that MIAMSIE alone will gain the traction required to meaningfully improve reporting across the MSI field. We suggest that widespread uptake will require a parallel intermediate option that can produce gradual improvements. Below, we suggest an abbreviated reporting standard called MSIcheck for this role. Other steps might include automated harvesting of the information requested by MIAMSIE and/or other standards [15]. This is possible for a significant subset of MIAMSIE but would require vendor support to achieve. Regardless of the discussion provided here, future work in this space should engage vendors, MSIS [27], as well as repositories (e.g., PRIDE [31] and metaspace2020 [34]) and journals.

The aim should be to introduce a successor standard that aggregates a clearly defined granular standard such as MIAMSIE with the requirements across the MSI field (McDonnell et al., MIAPE, imzML), achieves this by building on existing ontologies, and achieves measurable uptake by initially defining a more conservative and straightforward reporting expectation.

## MSIcheck

### Brevity with impact

**Figure 2:**
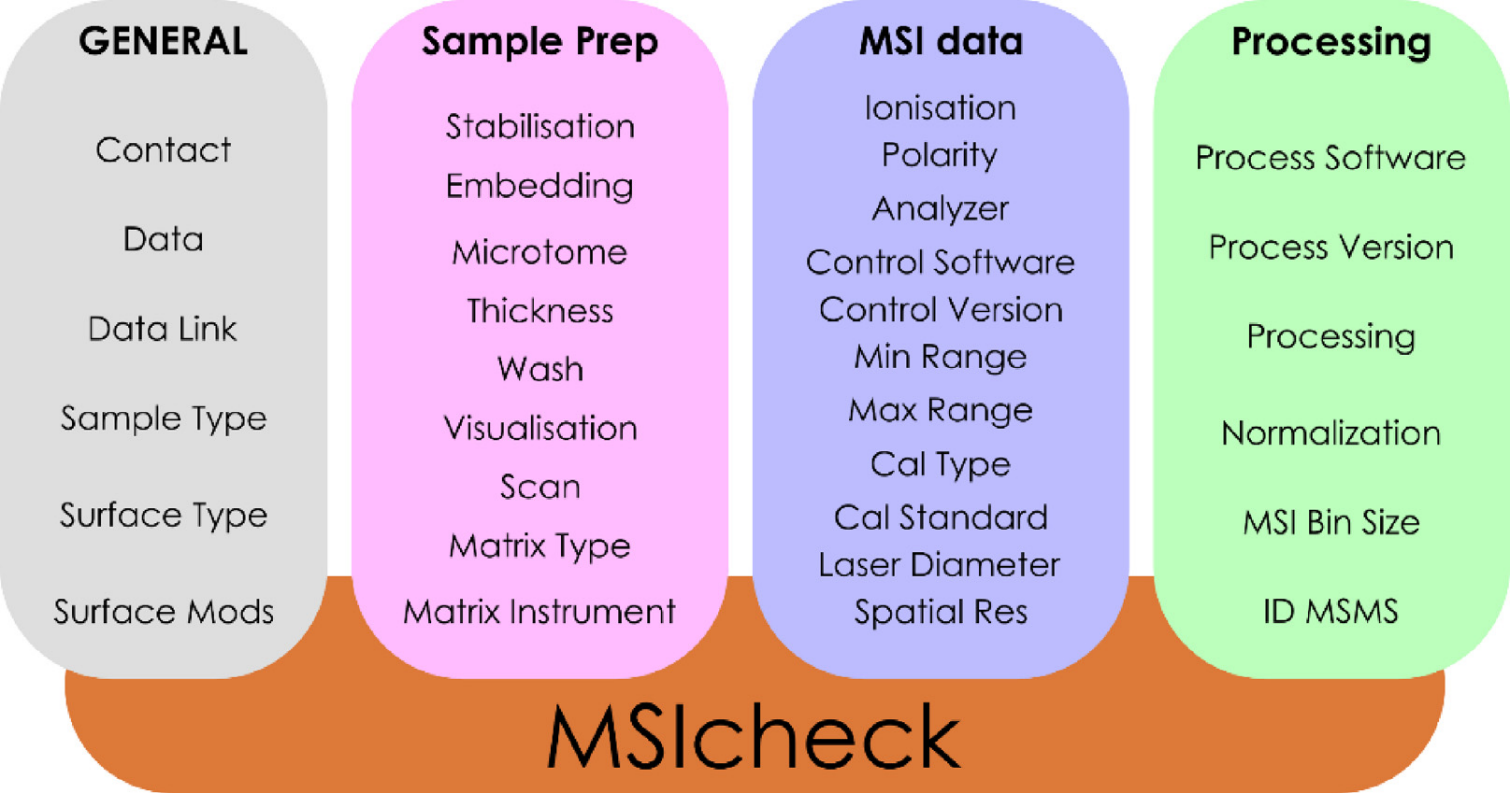
MSIcheck standard with the corresponding MIAMSIE field *Names*.

We believe that practical tools that support incremental change are effective mechanisms that can improve reporting and reproducibility [25]. This was the core motivation for creating MSIcheck: an abbreviated reporting template, based on MIAMSIE, that focuses on fields that we believe represent common problem points. The implication is that if these are addressed, a demonstrable and relatively rapid improvement to the field would result. The 32 MIAMSIE fields that comprise MSIcheck are presented in Fig. 2. Supplementary Table S5 provides the MIAMSIE *ID* number, *Name*, and *Description* for each reporting field. Notice that gating questions have been removed in a further effort to create a minimalist report format. The brevity of MSIcheck means it is easier to complete than the full MIAMSIE standard and, as a result, it has a greater number of practical applications. For example, MSIcheck can be used for editorial or peer reviews (similar to the Life Sciences Reporting Summary required for submission to *Nature* or the Minimum Standards of Reporting Checklist required for submission to *GigaScience*) or for *post-hoc* reviews of literature publications. MIAMSIE would be unsuitable for the majority of these use cases.

**Figure 3:**
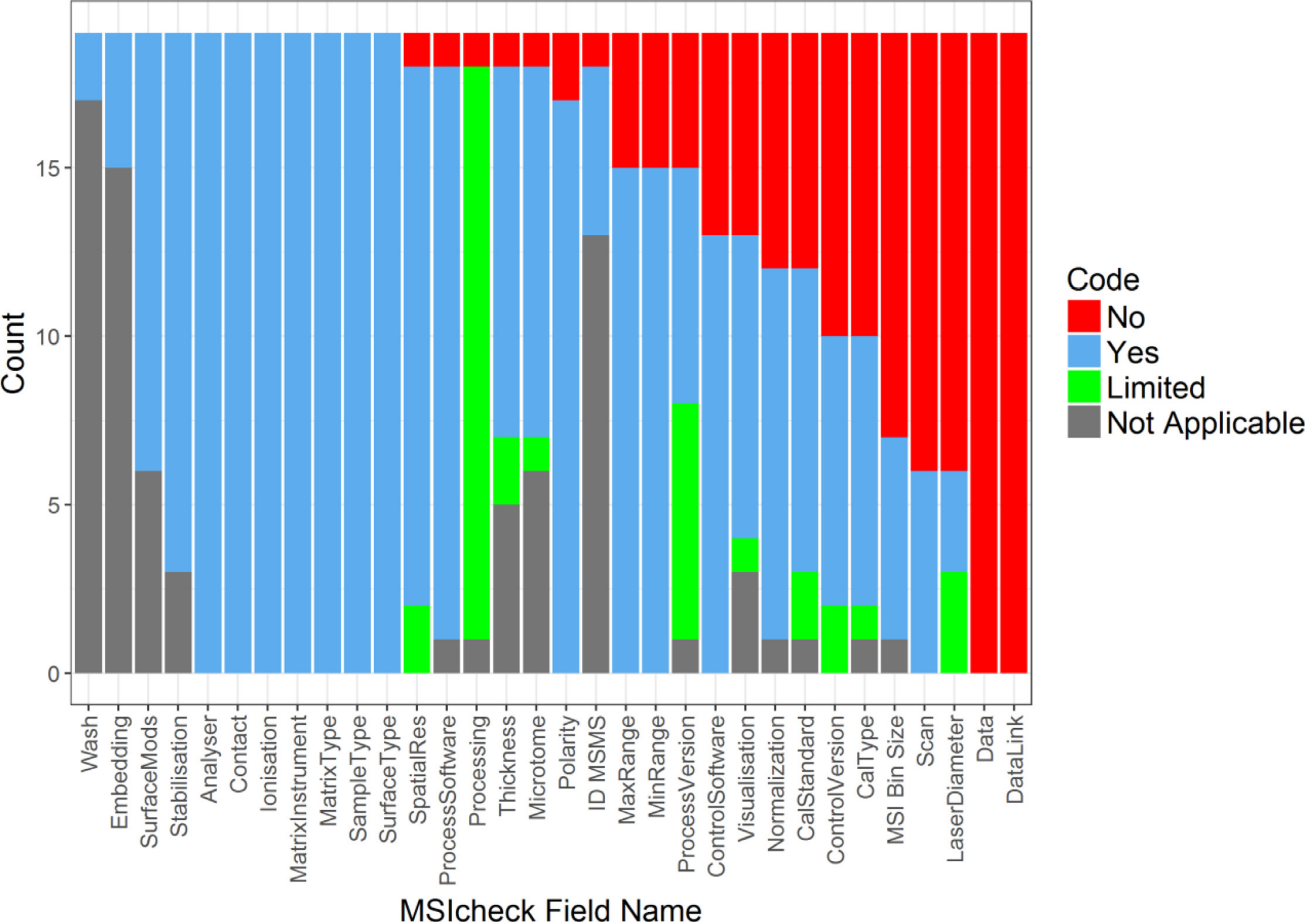
Counts for each MSIcheck field, indicating the number of articles (of 19) for which the field information was or was not provided (*Yes/No*), was *Limited* (more information required), or was not applicable to the article (*N/A*). Figure generated using R [29, 57-59]. See Additional Files (Element S8) for R script details.

MSIcheck can also provide a quantitative insight into the state of reporting quality field wide, following application to a significant subset of the published MSI literature. This is achieved by ensuring standardized data entry for MSIcheck fields in a readily accessible file format (e.g., ASCII) that subsequently allows import, transformation, and presentation of the data using open-source platforms (e.g., R [29]). Naturally, applying it to the entire MSI literature is unrealistic and beyond the scope of this work, and so here we applied MSIcheck to a one-month snapshot from 2017. This approach could regularly capture the state-of-the-field with respect to the direction and composition of MSI in a more transparent way than anonymous surveys [35] but also highlight progress made in addressing key perceived issues in MSI. These issues include, but are not limited to, data provision and the complete reporting of both software versions and processing pipelines. If used on a regular basis for a large enough portion of the literature, MSIcheck could track uptake over time and inform its own further development by presenting quantifiable metrics (see Fig. 3) that indicate which fields are poorly reported and should become focal points for improvement. Beneficial consequences of this may include the gradual uptake and growth of MSIcheck to become a complete standard (MIAMSIE), an annual publication reviewing the state of MSI, and the creation of a culture in which adherence to any standard could be advertised with a badge of reputability [10].

### Application of MSIcheck

With MSIcheck defined, we applied it to a review of two example studies from the laboratory of the last author (P.H.) as well as a retrospective review of the literature.

Similar to journal checklists, MSIcheck allows for rapid nonexhaustive publication assessment. Application here to two specific previous publications [36, 37] (Supplementary Table S6) highlighted that MSIcheck can be completed in less than 10 minutes, with time noted as the most important factor. Secondary, but still important, was the inclusion of MSIcheck for journal article reviewers. MSIcheck was considered comprehensive enough in a technical sense, as most, if not all, technical information of immediate import is there. Not unexpectedly, there was also interest in the subjective inclusion of other MIAMSIE fields in MSIcheck, e.g., including the *Hypothesis* of a study or its major outcomes. While adding too many fields would make MSIcheck more time consuming to complete, our experience is that a first author−driven MIAMSIE review can be achieved in less than 20 minutes.

For the literature MSIcheck, all articles discoverable in PubMed using the search terms *maldi* and *imaging* were selected for the month of June (2017). Manual evaluation of these 31 documents resulted in a final list of 19 primary MSI research articles that were readily accessible (i.e., not behind a paywall) and excluded reviews, protocols, and opinion pieces. The full list of articles and their inclusion status is provided in Supplementary Table S7. The 19 articles selected were reviewed using MSIcheck [38-56]. To avoid assessing the quality of the underlying science, which is not the intended purpose of MSIcheck, the review was limited to the abstract, materials and methods, as well as the supplementary information, as this can often contain an extended methods sections. The focus was ultimately if the information required by each field was included, rather than on the information itself. As such, the fields were placed into one of four categories summarizing the degree to which the information was included in the paper. These categories were *“Yes”* (the information was provided), *“No”* (the information was not provided), *“Limited”* (the information was partially provided but more detail was required), and *“Not Applicable”* (the field was deemed to be not relevant, e.g., a direct MSI analysis paper may not use an *in situ* chemistry, or *in situ* MS/MS may not have been required). An assumption made here is that subjective evaluation of the importance of providing some information vs some information not being applicable (*N/A*) will not greatly impact the final assessment of the publications reviewed. It is not possible, even with curation, to perfectly address the subjective interpretation of whether a study has provided only limited information for a particular field or has provided the information requested. In short, a perfect *post-hoc* assessment is difficult to achieve; however, as much care as practical was taken during review. The potential for misinterpretation of what fields are required or not applicable is a strong argument for standardization. If a field is needed, it will be completed in the standard; there is reduced ambiguity in filling out a community-accepted granular reporting schema. Note also that some of the current MSIcheck fields would normally require sample or dataset disambiguation; they may have different values for different subsets of the data, and this would need to be noted. As the focus of MSIcheck was to evaluate if the information required was included in the paper or not, these multiple values could conveniently be collapsed into a single value for each field; either the information was provided for all subsets of the data (a value of *“Yes”*) or it was provided for some subsets of the data and not others (a value of *“Limited”*).

An overview of the retrospective MSIcheck results are presented in Fig. 3, as a count of the *Yes*, *No*, *Limited*, and *N/A* responses. There are multiple noteworthy conclusions to be drawn from this output. These are discussed in more detail in the following sections.

### General observations from the one-month MSI review

There were noteworthy trends apparent from the MSIcheck summary in Fig. 3. First, several fields were reported well, including many *Sample Preparation* and *MSI Data* category fields such as *Analyzer, Ionization, and Matrix*. The fields that were consistently not well reported included *Scan, Laser Diameter*, as well as *Cal Type, Cal Standard*, *Control Version*, and *Process Version*. Scan resolution determines the accuracy of optical alignment to sample stage position prior to MSI data acquisition and consequently impacts the quality of surface region selection for MSI. Laser diameter impacts not only the spatial resolution of MSI but the appropriate use of oversampling. Oversampling can be inferred if both spatial resolution and laser diameter are reported; this is not the norm. The poor reporting of calibration is typically confined to those studies that employ MS instruments that decouple ionization and mass analysis (fourier transform mass spectrometry, FTMS). These instruments do not always require calibration prior to each measurement, and as such, this information is easily overlooked. Finally, MS control and MSI processing software version were not well reported. This impacts methods reproducibility, particularly as many software packages are readily updated or patched, which can alter how they perform processing or analysis steps. To use the example of FTMS, the latest versions of ftmsControl (solarix instruments) enable filtering for significant data reduction and also remove artifacts introduced by the signal processing (i.e., Fourier transform). Such steps need crystal-clear definition, as they impact what is being considered the “raw” output of an MS platform prior to any user processing and analysis. While this is complicated by the proprietary nature of some hardware and software, the broad-strokes definition of all data acquisition and preprocessing steps should be available. If not, where are these details being recorded and how can the appropriateness of these processes be critiqued either now or in the future? This question is growing more relevant as subscription-based sales models for analysis software become more common. If software can be updated or altered without the user being able to return to a prior version, methods reproducibility becomes even harder to achieve.

Observations that relate to day-to-day implementation became obvious following the MSIcheck literature review. First, there are fields for which the high N/A count supports the modification of MSIcheck to remove these fields. A good example is that not many of the publications modified their surfaces (*Surface Mods*). As suggested above, a further review of a much larger number of articles (6 months–1 year) could allow modification of MSIcheck, based on the quantitative assessment resulting from such a review (see Fig. 3). The impact of a regular bird's-eye view of MSI reporting is difficult to predict. However, we anticipate that this information will provide an incentive to be a visible contributor to improving the field [20]. We certainly want to demonstrate a commitment to scientific rigor and allow for more informed decisions regarding the design and content of a community-derived high-impact reporting standard.

Finally, the initial MSIcheck review was performed by two of the authors. A comparison of these independent reviews revealed some inconsistencies in the responses. Qualitatively, the reviews had a high degree of overlap, and this was mirrored in the focused review (Supplementary Table S6). The observation that not all answers were similar highlights that even with a prompting review system, results still need to be curated, as is the case with repositories such as PRIDE [60].

### “Show us the data”

Providing raw data allows other researchers to reproduce results, apply methods or conclusions in subsequent studies, improve on workflows, and make comparisons with existing studies [11, 61]. Often, the only way to validate methods reproducibility is to apply reported methods to the raw data. The immediate issue is that none of the articles reviewed by MSIcheck provide direct access to raw data; this observation was mirrored in the MIAMSIE reviews (Supplementary Tables S2 and S3).

Using MSIcheck as is, there are two caveats that may indicate that data availability was overlooked. First, data links could have been included in obscure or unreviewed locations or could have been made available as separate links by the journal or publisher. Links should be provided in a prominent and highly visible location, both in the article and online. The Data Availability Statement that appears in a side bar on the first page of articles published by *PLOS ONE* is a good example of such a prominent location. Second, raw data submission to a repository may have been delayed until after publication. In this scenario, the data resource would obviously not be cited in the preceding publication but would exist as its own citable resource.

Assuming the review is correct, its results contrast with the trend of increased data sharing in other MS-specific fields such as proteomics [62]. From a practical standpoint, the fact that raw data makes it easier to discover novel phenomena, draw alternative conclusions, and correct mistakes is secondary to incentives and ease of data provision [20, 63]. Current incentives should outweigh apprehensions surrounding re-use, and ease of data provision will only increase as both infrastructure and the processes supporting streamlined and efficient data provision and analysis are developed further [64, 65].

Apprehension surrounding data provision may stem from the discovery of errors, which impacts reputation, or the potential loss of future publications. First, although embarrassing, mistakes and analytical errors do happen and should be corrected quickly. Rather than stoking fears, raw data provision supports the contention that discovery of conflicting findings is welcomed, that any mistakes are honest, and ultimately that authors value their legacy in the literature [66].

Second, it is possible that novel or noteworthy findings can be identified through the re-use of data; this is but another avenue toward the cumulative gain of knowledge [64, 65]. In fact, bleeding-edge research relies on repository data. Development of MSI data analysis methods, e.g., uses such data to compare the results of novel methods against published results using the same data. Unfortunately, most independent groups do not have the time or resources for further in-depth analysis of repository datasets, particularly due to the need for specialist informaticians for “big-data” mining (e.g., multi-omics, systems biology). This is championed by consortia (e.g., the Human Proteome Project [67], European Molecular Biology Laboratory [EMBL] [68]) and may thus explain the 88% (of >2M total) of data citation index (DCI) entries on Web of Science that are uncited, despite the bulk of DCI entries representing the “hard” sciences, including PRIDE entries [69-71]. Nevertheless, more than 160 TB of data were downloaded from PRIDE in 2014 [72]. To put this in context, the total size of PRIDE reached 100 TB in 2015 [62]. MSI data generation alone was estimated at >1 TB/day globally in 2015 [35]. Thus, in general, MS datasets are being re-used, but the scope of this re-use globally is still small. The idea that other groups are waiting to “poach” these datasets is not realistic or productive. In combination with data citations (or digital object identifiers, DOIs [72]), which make datasets citable objects in the literature, re-use should not be an issue. This is supported by the original laboratories being best placed to re-analyze their data in context and considering that the current publish-or-perish culture devalues re-analysis and reproduction [20]. Ultimately, until data provision becomes the MSI standard, the pragmatist's approach is to make re-use possible in principle by providing raw data in order to emphasize the importance of data citation and to focus first on achievable analyses and spearhead subsequent re-analyses in collaboration with colleagues in complementary fields.

Apprehensions aside, ease of completion/provision and incentives [20] are big determinants of data provision [20, 63]. Fortunately, there are solutions in place or under development that standardize and incentivize MSI data sharing. This includes suitable repositories, as MSI generates data of significant size (>100 GB for FTMS) and in unique spatially referenced formats. Raw MSI data-sharing was made feasible both by the introduction of a shared community data format, imzML [30], as well as the modification of the PRIDE repository in 2015 to accept both imzML and vendor formats [73]. This was significant as PRIDE can support and curate large datasets, maintain privacy of the data until manuscript acceptance, and is part of the ProteomeXchange consortium, which integrates MS data from multiple repositories [62]. Although this is a concrete step in the right direction, as of 5 February 2018, the PRIDE repository only contained nine datasets of experiment-type MSI. PRIDE seems underutilized, which may suggest issues surrounding data upload. This absence may also be explained by more active engagement with the Metabolights repository [74] that, like PRIDE, is hosted by EMBL-European Bioinformatics Institute. This resource has become a popular alternative; 326 datasets were available using the search term “mass spectrometry imaging” and filtering for “study” (10 July 2018). This result is perhaps not surprising given the skew in MSI toward metabolite imaging.

Equally important to the availability of repositories is the incentive to upload. In this context, MSI data availability is also being addressed by additional consortia such as Metaspace2020 [35, 75] and software providers such as SCiLS (SCiLS Cloud [76]). Similar to PRIDE, Metaspace supports large dataset uploads for high mass resolution MSI data (typically FTMS), directly through a drag-and-drop web interface. However, it is currently a resource with a fundamentally different purpose to PRIDE. Metaspace processes, annotates [75], and presents MSI data (see summary [34]) but does not provide access to the raw data. There is therefore a strong incentive for data upload, as researchers can easily benefit from access to an MSI-specific annotation tool. PRIDE was only modified to allow partial MSI upload and does not generate MSI DOIs [72], supported by a large consortium of academia (e.g., EMBL) and industry (e.g., MS vendors, journals), while only sharing the processed MSI output data in the form of ion intensity maps. The combination of simple interface, access to annotation tools, and removal of apprehensions surrounding raw data provision may explain the rapid uptake of Metaspace, which grew from 1,433 (5 July 2017) to 2,249 (29 January 2018) complete datasets in the space of approximately seven months. This is in contrast to the 4,657 publicly discoverable datasets on PRIDE (29 January 2018), of which 9 were MSI. Metaspace has therefore exhibited a growth rate ($ {\sim} \, 136$ datasets/month) similar to that of PRIDE in 2015 (average of 150 datasets/month) [72]. The MSI community should strive to achieve raw data provision rates similar to that seen for Metaspace. The future possibility of authors permitting raw data download from Metaspace should not be discounted, nor should PRIDE, which can accept MSI data, has extensibility to allow the provision of additional reporting attributes (MIAMSIE) from controlled vocabularies [31] and may one day offer complete MSI submissions that are completely integrated with PX/PRIDE workflows [64, 65].

With the indexation of data in repositories (DCI), citability of data objects (DOIs), continuing development of repository infrastructure and tools, as well as the expectation of data provision by journals, funding bodies, and major institutions, data sharing is becoming an inescapable but ultimately win-win situation [64]. The underlying principle is that if it is easier for other researchers to use your data, they will do so more frequently, cite your data and publications more often [65], increase their individual impacts, and improve your h-index. In line with these findings, and assuming the case for raw data provision in MSI can be supported by monitoring (MSIcheck), it would be beneficial to examine the re-use rates and citation metrics for random selections of primary MSI articles that do and do not provide access to raw data; the expectation being that raw data provides a citation advantage [77].

## Conclusions

At the core of healthy peer-reviewed scientific literature is the ability to both critique and build upon the work of other researchers. In order for that to be possible, methods, results, and data must be reported to a standard that allows for them to be reproduced and hence for their significance in the wider context of the field to be given its appropriate weight. Here, we have presented our contribution to the discussion regarding a reporting standard for MSI. MIAMSIE provides content and a suggested format that could eventually, with field-wide discussion and development, become a standard sufficient to guarantee reporting quality, in the same spirit as MIRAGE and the granular MSI standard suggested by McDonnell [23]. MSIcheck concedes the difficulty in promoting uptake by providing an easy-to-complete abbreviated reporting standard that can be used in different ways to improve not only reporting of experiments but also aid in peer review, literature reviews, and the establishment of reporting reputation as a tangible incentive to stimulate engagement of all MSI researchers with community-endorsed reporting standards.

As demonstrated by MSIcheck, true raw data availability has yet to become a stage-gate for MSI reporting. In addition, there appear to be significant improvements to be made in reporting of both analysis/processing steps and for MS-specific calibration metrics. This is an unfortunate *status quo*. We believe true change will be underpinned by our capacity to measure, report on, and promote the provision of data across the entire MSI field, in line with efforts across the sciences to create an open data culture. Finally, it is imperative for groups that excel in reporting to drive the development of tools that simplify the reporting of our increasingly complex experiments and to show the way by implementing these tools in conjunction with both repositories and publishers. Ultimately, standards such as MIAMSIE are useful despite the lack of data provision in the field, particularly as diagnostics and for *in-house*record keeping. Time will tell how useful the described approach will be and how well it will translate to improvements in reporting and reproducibility for MSI.

## Table of Contents (TOC) Figure

**Figure:**
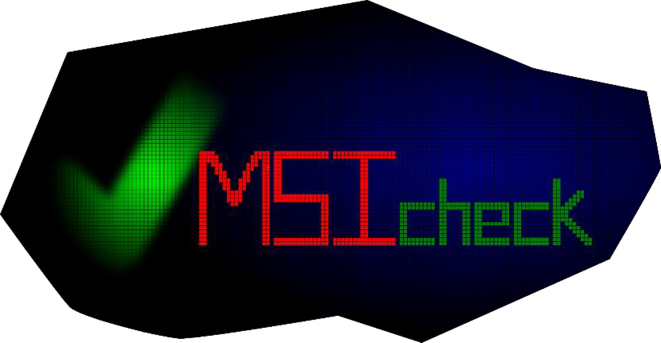


## Availability of supporting data

All data and material are available in the main text or as Additional Files.

## Additional files

**Additional Table S1**. The MIAMSIE standard template (no gating functionality)

**Additional Table S2**. Application of first generation MIAMSIE to post-publication report for references Boughton and Hamilton (2017), Jarvis *et al*. (2017) and Andersen *et al*. (2017)

**Additional Table S3**. Application of first generation MIAMSIE to post-publication report for reference Undheim *et al*. (2015)

**Additional Table S4**. Application of first generation MIAMSIE to data-set report

**Additional Table S5**. MSIcheck standard with the corresponding MIAMSIE ID, Name and Description provided

**Additional Table S6**. Research leader MSIcheck (*Yes/No/NA/Limited*) of two publications

**Additional Table S7**. Papers found using the PubMed search string *“((“2017/5/1”[Date—Publication] : “2017/6/1”[Date—Publication])) AND maldi AND imaging”* on the 3rd of July 2017

**Additional File S8**. R script required for plotting of MSIcheck review results

## Abbreviations

DCI: data citation index; DOI: digital object identifier; EMBL: European Molecular Biology Laboratory; FTMS: Fourier Transform Mass Spectrometry; GUI: graphical user interface; LDI: laser desorption/ionization; MALDI: matrix-assisted laser desorption/ionization; MIAMSIE: Minimum Information About a Mass Spectrometry Imaging Experiment; MIAPE: Minimum Information About a Proteomics Experiment; MIRAGE: Minimum Information Required for a Glycomics Experiment; MS: mass spectrometry; MSI: mass spectrometry imaging; MSIS: Mass Spectrometry Imaging Society; SIMS: secondary ion mass spectrometry.

## Competing interests

The authors declare that they have no competing interests.

## Funding

O.J.R.G.'s position was funded by the Australian Research Council (ARC) Centre of Excellence for Convergent Bio-Nano Science & Technology (CBNS, ARC Project CE140100036). L.J.W.'s position was funded through Bioplatforms Australia (Australian Government). E.A.B.U. was funded by the ARC (DE160101142).

## Author contributions

**Table:** 

	O.J.R.G.	L.J.W.	M.R.C.	B.A.B.	B.H.	E.A.B.U.	M.B.	P.H.
Conceptualization	Lead	Equal	Equal					
Data curation	Equal	Equal	Equal					
Formal analysis	Equal	Equal	Supp.	Supp.	Supp.	Supp.	Supp.	
Validation	Equal		Supp.	Supp.	Supp.	Supp.	Supp.	Supp.
Writing (original)	Lead	Equal	Supp.					
Writing (editing)	Lead	Equal	Supp.	Supp.	Supp.	Supp.	Supp.	Supp.

## Supplementary Material

Supplemental File
