# Supplementary material for: Balancing sufficiency and impact in reporting standards for mass spectrometry imaging experiments
Source: Gigascience. 2018 Aug 14;7(10):giy102. doi: 10.1093/gigascience/giy102 (PMC6203951; doi:10.1093/gigascience/giy102)
Supplement: Supplemental File [file giy102_supplemental_file.docx]

Supplementary Information

Balancing sufficiency and impact in the reporting of mass spectrometry imaging experiments

Ove J. R. Gustafsson^1,2^, Lyron J. Winderbaum^2^, Mark R. Condina^2^, Berin A. Boughton^3^, Brett R. Hamilton^4,5^, Eivind A. B. Undheim^5^, Michael Becker^6^, and Peter Hoffmann^2^

1 ARC Centre of Excellence in Convergent Bio-Nano Science & Technology (CBNS), University of South Australia, Mawson Lakes, South Australia 5095, Australia

2 Future Industries Institute, University of South Australia, Mawson Lakes, South Australia 5095, Australia

3 Metabolomics Australia, School of BioSciences, University of Melbourne, Parkville, Victoria 3010, Australia

4 Centre for Microscopy and Microanalysis, University of Queensland, St. Lucia, Queensland 4072, Australia

5 Centre for Advanced Imaging, University of Queensland, St. Lucia, Queensland 4072, Australia

6 Boehringer Ingelheim Pharma GmbH & Co. KG, Ingelheim am Rhein 55216, Germany

**Table S1.** The MIAMSIE standard template (no gating functionality)

| *No.* | *Field name* | *Description* | *Category* | *Valid values* | *Value* |
| --- | --- | --- | --- | --- | --- |
| 1 | Contact | Stable (ideally *long term*) contact information for experimenter(s) | General | Email, ... |  |
| 2 | Organizational Unit | The organizational unit or laboratory where the experiment was conducted | General | Future Industries Institute, ... |  |
| 3 | Institution | The institution(s) where the experimental data collection was completed | General | The University of South Australia, ... |  |
| 4 | Title | Single sentence experiment title (e.g. publication title) | General | MALDI imaging of glycans in a murine kidney, ... |  |
| 5 | Aim | Experimental aim(s) | General | To demonstrate that... |  |
| 6 | Hypothesis | Experimental hypothesis(es) | General | NA, ... |  |
| 7 | Data | Can data be downloaded without restriction from a repository (PRIDE) or viewed on a Cloud platform (SCiLS Cloud, metaspace2020)? | General | Completely Available, Partially Available, Not Available |  |
| 8 | Data Negative | Have any datasets that showed “negative results” been included with the reported data? | General | Yes, No |  |
| 9 | Data Link | URL, link or resource identiﬁer for the experimental data | General | ebi.ac.uk/pride/archive/projects/ PXD004018, ... |  |
| 10 | Data Format | Raw data ﬁle format (imzML is mass spectrometry imaging (MSI) community standard format) | General | imzML, Thermo RAW, ... |  |
| 11 | Data Files | A list of the ﬁles available at the link provided in MIAMSIE 9(Data Link) | General | Section1.imzml, Section1.ibd, Section2.imzml, Section2.ibd, LC.RAW, ... |  |
| 12 | Dataset Names | A list of dataset names, and the ﬁles in which they can be found | General | Region1.1 (Section1.imzml, Section1.ibd), Region1.2 (Section1.imzml, Section1.ibd), Region2.1 (Section1.imzml, Section1.ibd), Region2.2 (Section1.imzml, Section1.ibd), Region2.3 (Section1.imzml, Section1.ibd), liquid chromatography (LC) (LC.RAW), ... |  |
| 13 | Sample Type | The type of sample | General | Animal model organ, clinical, geological, ... |  |
| 14 | Sample List | A list of samples, and if possible (i.e. if MIAMSIE 7 (Data): Completely Available) the datasets associated to them | General | Mouse 1 (Region 1.1, Region 2.1), Mouse 2 (Region 1.1, Region 2.2, Region 2.3), Mouse 3 (Region 1.2), ... |  |
| 15 | Sample Disambiguation | If multiple samples are in a single dataset, information distinguishing the data from diﬀerent samples | General | NA, Region 1.1 contains tissue from both Mouse 1 and Mouse 2 and these consist of disjoint acquisition regions clearly visible from the spatial distribution (X-Y coordinates of the spectra) — the long and thin region is that from Mouse 1 while the round region that of Mouse 2, ... |  |
| 16 | Surface Type | Surface analyzed or used as an underlying substrate for analysis | General | Indium tin oxide (ITO), glass, metal, nanostructured substrate, ... |  |
| 17 | Surface Mods | Was the surface analyzed modiﬁed in anyway? | General | NA, functionalized, coated, ... |  |
| 18 | Surface Sample | Was a surface directly measured by MSI? | General | NA, micro-organism culture, thin layer chromatography (TLC)-matrix assisted laser desorption/ionization (MALDI), ... |  |
| 19 | MS Model | The instrument model used to acquire mass spectra. | MSI data | ultrafleXtreme, autoflex, solarix, ... |  |
| 20 | MS Vendor | The mass spectrometer vendor. | MSI data | Bruker Daltonics, Thermo, Waters, Sciex, Shimadzu ... |  |
| 21 | Ionization | Ionization type used. | MSI data | MALDI, laser desorption/ionization (LDI), surface assisted laser desorption/ionization (SALDI), secondary ion mass spectrometry (SIMS), laser ablation electrospray ionization (LAESI), desorption electrospray ionization (DESI), ... |  |
| 22 | Polarity | Polarity (positive, negative) | MSI data | Negative, positive |  |
| 23 | MS Customization | Specific modifications or additions to MS instrument. | MSI data | NA, laser geometry, vacuum, ... |  |
| 24 | Voltages | Voltages used in the MS instrument (as relevant) [V]. | MSI data | Quadrupole, acceleration, reflectron, detectors, ... |  |
| 25 | Extraction | Was delayed extraction used? Value? (ns) | MSI data | NA, Yes, No, 200 ns, ... |  |
| 26 | Analyzer | Mass analyzer used. | MSI data | time-of-flight (ToF), quadrupole time-of-flight (Q-ToF), linear trap quadrupole (LTQ), Ion Trap, Orbitrap, Fourier  Transform ion cyclotron resonance (FTICR), ... |  |
| 27 | Reflectron | Was a reflectron used? | MSI data | NA, Yes, No |  |
| 28 | Mass Resolution | Estimate the mass resolution achieved at a defined *m/z (FWHM)* | MSI data | NA, 10k at *m/z* 500, ... |  |
| 29 | Detector | Type of detector used. | MSI data | Electron multiplier, position sensitive, ... |  |
| 30 | Post Acq Process | Was data processed during acquisition? | MSI data | NA, Yes, No, data reduction, peak picking, ... |  |
| 31 | Control Software | Name of MS control software. | MSI data | flexControl, ... |  |
| 32 | Control Version | Version of MS control software. | MSI data | NA, 3.0, ... |  |
| 33 | Min Range | Minimum *m/z* for the acquired mass range. | MSI data | 300, ... |  |
| 34 | Max Range | Maximum *m/z* for the acquired mass range. | MSI data | 2000, ... |  |
| 35 | Mass Window | Was a quadrupole or similar used to select *m/z* windows? | MSI data | NA, Yes, No, *m/z* 200-300, ... |  |
| 36 | Cal Type | Type of calibration employed. | MSI data | NA, internal, external, lock-mass, ... |  |
| 37 | Cal Standard | Calibration standard or standard mixture(s) used. | MSI data | NA, ... |  |
| 38 | Cal Min | Minimum *m/z* for range calibrated. | MSI data | NA, 300, ... |  |
| 39 | Cal Max | Maximum *m/z* for range calibrated. | MSI data | NA, 2000, ... |  |
| 40 | Speed | Speed of scan (if applicable). | MSI data | NA, ... |  |
| 41 | Clinical | Sample clinical origin. | Sample Prep | NA, resection, needle core biopsy, ... |  |
| 42 | Species | The species of the sample(s). | Sample Prep | NA, Homo sapiens, ... |  |
| 43 | Age | Age of sample(s) [days or years]. | Sample Prep | NA, days, years, ... |  |
| 44 | Gender | Gender of sample(s). | Sample Prep | NA, male, female, ... |  |
| 45 | Bio Sampling | Method of sample dissection. | Sample Prep | NA, surgical, punch biopsy, scalpel, ... |  |
| 46 | Post Mortem | Time in minutes prior to processing of post-mortem sample. | Sample Prep | NA, 1, 5, 10, ... |  |
| 47 | Species | The species of the botanical sample(s). | Sample Prep | NA, Hordeum spontaneum, ... |  |
| 48 | Botany Source | The source (location) of the botanical sample(s). | Sample Prep | NA, Australia, ... |  |
| 49 | Growth Info | Any specific information for the germination and / or growth of the plant(s). | Sample Prep | NA, ... |  |
| 50 | Material Type | The geological type and / or composition of the sample(s). | Sample Prep | NA, volcanic glass, ... |  |
| 51 | Geo Source | The source (location) of the geological sample(s). | Sample Prep | NA, Australia, ... |  |
| 52 | Base Material | The majority element / component of the sample(s). | Sample Prep | NA, iron, silicon, ... |  |
| 53 | Dopants Additives | Any dopants or specific additives in the sample(s). | Sample Prep | NA, boron, neodymium, ... |  |
| 54 | Synthesis | The synthetic process used to generate the sample(s). | Sample Prep | NA, ... |  |
| 55 | Stabilization | What stabilization method was used? | Sample Prep | NA, dehydration, heat inactivation, alcohol/cross-linking fixation, freezing, ... |  |
| 56 | Stabilization Type | What was the stabilization process and/or solution composition? | Sample Prep | NA, overnight formalin incubation, ... |  |
| 57 | Embedding | Embedding method for sample. | Sample Prep | NA, optimal cutting temperature (OCT), carboxymethyl cellulose (CMC), gelatin, paraffin, none, ... |  |
| 58 | Pre Storage | Sample storage time / conditions (pre-MSI sample preparation) [hours]? | Sample Prep | NA,... |  |
| 59 | Post Storage | Sample storage time / conditions (post-MSI sample preparation) [hours]? | Sample Prep | NA, ... |  |
| 60 | Orientation Sample | Orientation of sample in block. | Sample Prep | NA, transverse, longitudinal, ... |  |
| 61 | Orientation Section | Orientation and / or location of sections on analytical surface. | Sample Prep | NA, ... |  |
| 62 | Microtome | (Cryo)-microtome instrument used to section material. | Sample Prep | NA, ... |  |
| 63 | Microtome Temp | (Cryo)-microtome temperature used (⁰C). | Sample Prep | NA, ... |  |
| 64 | Mounting | Mounting method for sectioned material. | Sample Prep | NA, thaw mount, water mount, ... |  |
| 65 | Thickness | Tissue section thickness (μm). | Sample Prep | NA, 4, 6, 10, ... |  |
| 66 | Section Storage | Any storage of section (pre- or post-treatment)? | Sample Prep | NA, fridge, freezer, vacuum, inert gas, ... |  |
| 67 | Wash | Wash or solution treatment for section(s) and / or surface(s). | Sample Prep | NA, tissue fixation (native), re-hydration (alcohol-fixed) or Antigen Retrieval (formalin-fixed), ... |  |
| 68 | Misc Treatment | Miscellaneous treatment(s). | Sample Prep | NA, freeze-drying, UV irradiation, oven incubation, ... |  |
| 69 | Visualization | The visualization or histological staining method applied to the sample. | Sample Prep | NA, haematoxylin & eosin (H&E), scanning, photography, ... |  |
| 70 | Microscopy | Was microscopy used to visualize the sample? Magnification? | Sample Prep | NA, NanoZoomer 20x objective, ... |  |
| 71 | Scan | Was sample / section scanned prior to analysis for co-registration? | Sample Prep | NA, Yes, No |  |
| 72 | Scan Res | Resolution (dpi) of optical sample scan. | Sample Prep | NA, 2400, ... |  |
| 73 | Scan Instrument | Type of scanner used to acquire optical sample / section images. | Sample Prep | NA, flatbed scanner, ... |  |
| 74 | Scan Type | Same or adjacent section scanned? | Sample Prep | NA, same, adjacent |  |
| 75 | *In Situ* Chem Type | What *in situ* chemistry was used? | Sample Prep | NA, proteolytic, glycosidase, ... |  |
| 76 | *In Situ* Instrument | Instrument used for *in situ* chemistry. | Sample Prep | NA, ImagePrep, ChIP-1000, ... |  |
| 77 | *In Situ* App | *In situ* chemistry application method. | Sample Prep | NA, spray, sublimation, printing, ... |  |
| 78 | *In Situ* Reagent | Reagent concentration (M). | Sample Prep | NA, concentration of enzyme, derivatising agent, ... |  |
| 79 | *In Situ* Solution | Composition of solution(s) used for *in situ* chemistry (M). | Sample Prep | NA, 25 mM NH_4_HCO_3_, ... |  |
| 80 | Matrix | Was a “matrix” used to mediate ionization? | Sample prep | NA, Yes, No |  |
| 81 | Matrix Type | What compound or material was used to mediate ionization? | Sample Prep | NA, none, chemical, metal, nanoparticles, other, ... |  |
| 82 | Matrix Instrument | Instrument used for matrix preparation. | Sample Prep | NA, Manual sprayer, ImagePrep, sublimation chamber, iMatrixSpray, TM-sprayer, ... |  |
| 83 | Matrix App | Matrix application method. | Sample Prep | NA, spray, sublimation, printing, ... |  |
| 84 | Matrix Amount | Matrix concentration (mg/mL) or amount (mg, or mg/unit area). | Sample Prep | NA, 20 mg/mL, 20 mg, ... |  |
| 85 | Matrix Solvent | Solvent composition (% v/v). | Sample Prep | NA, 50% acetonitrile (ACN) with 0.2% trifluoroacetic acid (TFA), ... |  |
| 86 | Shots | Shots acquired per position. | MSI data | NA, 200, ... |  |
| 87 | Laser Type | The type and model of laser system as well as wavelength. | MSI data | NA, Smartbeam II (355 nm), ... |  |
| 88 | Laser Diameter | Laser spot size, or measured area (μm). | MSI data | NA, 20 μm, ... |  |
| 89 | Laser Repetition | Repetition rate of laser system. | MSI data | NA, 2 kHz, ... |  |
| 90 | ToF Nano | Was ToF-SIMS or nanoSIMS used? | MSI data | NA, ToF-SIMS, nanoSIMS |  |
| 91 | Ion Beam Type | What primary ion beam was used? | MSI data | NA, Au^+^, Au^3+^, C60^+^, Ar^+^, ... |  |
| 92 | Dynamic Static | What ionization regime was used? | MSI data | NA, dynamic, static |  |
| 93 | Align | Alignment method for MSI and visualization(s). | MSI data | flexImaging co-registration, ... |  |
| 94 | Spatial Res | The size of the data pixels (spectra) [µm]. | MSI data | NA, 20 µm, ... |  |
| 95 | Oversampling | Was oversampling used? | MSI data | NA, Yes, No |  |
| 96 | Sampling Fraction | Was laser rastered within a pixel / spectrum point? | MSI data | NA, Yes, No, 10 x 5 µm laser spot and ablated within a 50 x 50 µm spot |  |
| 97 | Scan Type | Scan line orientation. | MSI data | NA, horizontal line scan, vertical line scan, random, ... |  |
| 98 | Scan Pattern | Scan pattern for this line orientation. | MSI data | NA, flyback, meandering, or random access, ... |  |
| 99 | Scan Order | Scan order for spectra within lines. | MSI data | NA, bottom up, left right, right left, top down, ... |  |
| 100 | Scan Line Order | Scan order for lines. | MSI data | NA, bottom up, left right, right left, top down, ... |  |
| 101 | Delocalization | Was analyte delocalization examined / considered? | MSI data | NA, Yes, No |  |
| 102 | Process Software | Name of pre-processing software. | Processing | NA, flexAnalysis, ... |  |
| 103 | Process Version | Version of pre-processing software. | Processing | NA, ... |  |
| 104 | Processing | List of the pre-processing steps / pipeline used on the raw data. | Processing | NA, ... |  |
| 105 | Noise Estimation | How was noise estimated in the spectra? | Processing | NA, ... |  |
| 106 | Baseline Sub | Was baseline subtraction applied? | Processing | NA, Yes, No |  |
| 107 | Baseline Sub Type | Method of spectral baseline subtraction. | Processing | NA, TopHat, ... |  |
| 108 | Smooth | Was spectral smoothing applied? | Processing | NA, Yes, No |  |
| 109 | Smooth Type | Method of spectral smoothing. | Processing | NA, Gaussian, Savitzky-Golay, ... |  |
| 110 | Smooth Width | Width of smoothing kernel applied to spectra. | Processing | NA, ... |  |
| 111 | Smooth Other | Any other relevant settings for smoothing. | Processing | NA, number of cycles, ... |  |
| 112 | Normalization | Method used for intensity normalization. | Processing | NA, none, total ion count (TIC), root mean square (RMS), ... |  |
| 113 | Peak Picking | Were peaks picked? | Processing | NA, Yes, No |  |
| 114 | Peak Picking Type | Method of peak picking. | Processing | NA, centroid, isotopic-envelope, ... |  |
| 115 | SNR | Signal-to-noise (SNR) threshold employed for peak picking. | Processing | NA, 5, 10, ... |  |
| 116 | Peak Picking Other | Any other relevant settings for peak picking. | Processing | NA, maximum number of peaks, ... |  |
| 117 | Analysis Software | Name of analysis software. | Processing | NA, flexImaging, SCiLS Lab, ... |  |
| 118 | Analysis Version | Version of analysis software. | Processing | NA, ... |  |
| 119 | MSI Peak Eval | Evaluation method for peaks. | Processing | NA, Height, area, ... |  |
| 120 | MSI Bin Size | Mass filter width / range or bin size (*m/z*) for plotted ion images. | Processing | NA, 0.2 *m/z*, FTICR variable, ... |  |
| 121 | MSI Measured Theo | Measured or theoretical centre used for ion image plotting range(s). | Processing | NA, Measured, theoretical |  |
| 122 | MSI Interpolation | Pixels / spectra interpolated? If so, what method was used? | Processing | NA, Yes, No, ... |  |
| 123 | Quant Method | Quantitation method employed. | Other | NA, internal standard, adjacent dilution  series, reference standard application, ... |  |
| 124 | Quant Reagents | Description of quantitation reagents (standards) | Other | NA, ... |  |
| 125 | Quant Procedure | Description of quantitation procedure (if applicable) | Other | NA, ... |  |
| 126 | ID Mz | *m/z* for compounds/ions of interest | Other | NA, ... |  |
| 127 | ID MSMS | Was MS/MS on tissue performed? Are spectra included? | Other | NA, Yes, No |  |
| 128 | ID Add | Were additional identification or characterization methods applied? | Other | NA, LC-MS / MS, NMR, ... |  |
| 129 | ID Mass Tol | If matched to theoretical *m/z*, what was the mass tolerance in *m/z* | Other | NA, ... |  |
| 130 | Other | Other information for which none of the above fields applies | Other | NA, ... |  |

**Table S2.** Application of first generation MIAMSIE to post-publication report for references Boughton and Hamilton (2017), Jarvis *et al.* (2017) and Andersen *et al.* (2017).

| *MIAMSIE ID no.* | *Field name* | *Boughton and Hamilton (2017)* | *Jarvis et al. (2017)* | *Andersen et al. (2017)* |
| --- | --- | --- | --- | --- |
| 1 | Researcher | Berin Boughton | Berin Boughton | Berin Boughton |
| 2 | Organizational Unit | Metabolomics Australia, School of BioSciences | Metabolomics Australia, School of BioSciences | Metabolomics Australia, School of BioSciences |
| 3 | Institution | The University of Melbourne | The University of Melbourne | The University of Melbourne |
| 4 | Ethics Approval | NA | NA | NA |
| 5 | Contact | [baboug@unimelb.edu.au](mailto:baboug@unimelb.edu.au) | [baboug@unimelb.edu.au](mailto:baboug@unimelb.edu.au) | [baboug@unimelb.edu.au](mailto:baboug@unimelb.edu.au) |
| 6 | Title | MALDI Imaging of Kangaroo Brain Lipids | The genome of Chenopodium quinoa | MALDI imaging of plant secondary metabolites |
| 7 | Aim | Demonstrate distribution of lipids in kangaroo cerebellum | To demonstrate the distribution of saponins in quinoa seed | To map the storage location of thapsigargin and analogues |
| 8 | Hypothesis | NA | Saponins located to seed coat | NA |
| 9 | Abstract | doi.org/10.1007/978-3-319-47656-8_12 | doi:10.1038/nature21370 | doi.org/10.1104/pp.16.00055 |
| 10 | Data | Partially Available – Metaspace | Not Available | Not Available |
| 11 | Data Negative | No | No | No |
| 12 | Data Link | annotate.metaspace2020.eu/#/  annotations?ds=2016-09-  21 16h07m23s&db =ChEBI&sort=-msm | NA | NA |
| 13 | Data Format | NA | NA | NA |
| 14 | Data Files | NA | NA | NA |
| 15 | Dataset Names | NA | NA | NA |
| 16 | Sample Type | Wild Type - Kangaroo (Macropus giganteus) - Brain (cerebellum) | Chenopodium quinoa seed (Quinoa) | Plant root |
| 17 | Sample List | NA | NA | NA |
| 18 | Sample Disambiguation | NA | NA | NA |
| 19 | Surface Type | Glass | Tape, conductive carbon tape, glass | Carbon tape on glass |
| 20 | Surface Mods | NA | NA | NA |
| 21 | Clinical | NA | NA | NA |
| 22 | Species | *Macropus giganteus* | *Chenopodium quinoa* seed (Quinoa) | *Thapsia garganica* |
| 23 | Age | NA | NA | NA |
| 24 | Gender | NA | NA | NA |
| 25 | Bio Sampling | Cryosection | NA | Section |
| 26 | Post Mortem | NA | NA | NA |
| 27 | Stabilization Type | Freezing | Drying | Freezing |
| 28 | Storage | -80°C, unknown | Room temperature, unknown | -80°C, unknown |
| 29 | Orientation | NA | NA | NA |
| 30 | Embedding | NA | CMC | NA |
| 31 | Microtome | Cryo-microtome | Cryo-microtome | Cryo-microtome |
| 32 | Mounting | Thaw mount | Tape mount (modified Kawamoto method), freeze-dry | Compression mount to carbon tape, freeze dry |
| 33 | Thickness | 15 | 50 | 35-50 |
| 34 | Wash | NA | NA | NA |
| 35 | Visualization | NA | NA | NA |
| 36 | Scan | Yes | Yes | Yes |
| 37 | Scan Res | 4800 | 4800 | 4800 |
| 38 | Scan Instrument | Flatbed scanner | Flatbed scanner | Flatbed scanner |
| 39 | Scan Type | Same | Same | Same |
| 40 | *InSitu* Chem Type | NA | NA | NA |
| 41 | *InSitu* Instrument | NA | NA | NA |
| 42 | *InSitu* App | NA | NA | NA |
| 43 | *InSitu* Reagent | NA | NA | NA |
| 44 | *InSitu* Solution | NA | NA | NA |
| 45 | MS Model | Solarix XR - 7T | Solarix XR - 7T | Solarix XR - 7T |
| 46 | MS Vendor | Bruker Daltonics | Bruker Daltonics | Bruker Daltonics |
| 47 | Ionization | MALDI | MALDI | MALDI |
| 48 | Polarity | Negative | Positive | Positive |
| 49 | MS Customization | NA | NA | NA |
| 50 | Voltages | NA | NA | NA |
| 51 | Extraction | NA | NA | NA |
| 52 | Analyzer | FTICR | FTICR | FTICR |
| 53 | Reflectron | NA | NA | NA |
| 54 | Detector | FTICR | FTICR | FTICR |
| 55 | Control Software | ftmsControl | ftmsControl | ftmsControl |
| 56 | Control Version | 2 | 2 | 2 |
| 57 | Min Range | 200 | 200 | 200 |
| 58 | Max Range | 3000 | 3000 | 3000 |
| 59 | Cal Type | External | External / Internal | External / Internal |
| 60 | Cal Standard | Red phosphorous clusters | Red phosphorous clusters / DHB clusters | Red phosphorous clusters / DHB clusters |
| 61 | Cal Min | 200 | 200 | 200 |
| 62 | Cal Max | 2000 | 2000 | 2000 |
| 63 | Shots | NA | NA | NA |
| 64 | Speed | 1 s | 2-3 s | 1 s |
| 65 | Matrix | 1,8-bis(pyrrolidinyl)naphthalene | 2,5-dihydroxybenzoic acid (DHB) | 2,5-dihydroxybenzoic acid (DHB) |
| 66 | Matrix Instrument | TM-sprayer | TM-sprayer | Sublimation chamber |
| 67 | Matrix App | Spray | Spray | Sublimation |
| 68 | Matrix Amount | 5 mg/mL | 40 mg/mL | 0.1-0.2 ng/mm^2^ |
| 69 | Matrix Solvent | Acetone (100%) | Methanol (70%) | NA |
| 70 | Laser Type | Smartbeam II | Smartbeam II | Smartbeam II |
| 71 | Laser Diameter | NA | NA | NA |
| 72 | Laser Repetition | 2 | 2 | 2 |
| 73 | Align | flexImaging co-registration | flexImaging co-registration | flexImaging co-registration |
| 74 | Spatial Res | 150 | 50 | 50-100 |
| 75 | Oversampling | No | No | No |
| 76 | Scan Type | Horizontal line scan (fastest) | Horizontal line scan (fastest) | Horizontal line scan (fastest) |
| 77 | Scan Pattern | NA | NA | NA |
| 78 | Scan Direction | Top down | Top down | Top down |
| 79 | Scan Line Direction | Left to Right | Left to Right | Left to Right |
| 80 | Delocalization | No | No | No |
| 81 | Process Software | ftmsControl | ftmsControl | ftmsControl |
| 82 | Process Version | 2 | 2 | 2 |
| 83 | Processing | NA | NA | NA |
| 84 | Noise Estimation | NA | NA | NA |
| 85 | Baseline Sub | NA | NA | NA |
| 86 | Baseline Sub Type | NA | NA | NA |
| 87 | Smooth | NA | NA | NA |
| 88 | Smooth Type | NA | NA | NA |
| 89 | Smooth Width | NA | NA | NA |
| 90 | Smooth Other | NA | NA | NA |
| 91 | Normalization | NA | NA | NA |
| 92 | Peak Picking | Yes | Yes | Yes |
| 93 | Peak Picking Type | Centroid | Centroid | Centroid |
| 94 | SNR | NA | NA | NA |
| 95 | Peak Picking Other | NA | NA | NA |
| 96 | Analysis Software | flexImaging / SCiLS Lab | flexImaging | flexImaging |
| 97 | Analysis Version | 4.1 / 2016a | 4.1 | 4.1 |
| 98 | MSI Peak Eval | Height | Height | Height |
| 99 | MSI Bin Size | Variable | Variable | Variable |
| 100 | MSI Measured Theo | Measured | Measured | Measured |
| 101 | MSI Interpolation | No | No | No |
| 102 | Quant Method | NA | NA | NA |
| 103 | Quant Reagents | NA | NA | NA |
| 104 | Quant Procedure | NA | NA | NA |
| 105 | ID Mz | Yes - Metaspace | *m/z* accurate mass match | *m/z* accurate mass match |
| 106 | ID MSMS | NA | NA | NA |
| 107 | ID LCMSMS | NA | LC-MS/MS | LC-MS/MS |
| 108 | ID Mass Tol | < 5 ppm | < 2 ppm | < 5 ppm |
| 109 | Other | NA | NA | NA |

**Table S3.** Application of first generation MIAMSIE to post-publication report for reference Undheim *et al.* (2015).

| *MIAMSIE ID no.* | *Field name* | *Reference Undheim et al. (2015)* |
| --- | --- | --- |
| 1 | Researcher | Brett Hamilton, Eivind A B Undheim |
| 2 | Organizational Unit | Centre for Advanced Imaging; Centre for Microscopy and Microanalysis |
| 3 | Institution | The University of Queensland |
| 4 | Ethics Approval | NA |
| 5 | Contact | [b.hamilton@uq.edu.au](mailto:b.hamilton@uq.edu.au) |
| 6 | Title | MALDI MSI of centipede venom glands |
| 7 | Aim | To assess the distribution of toxins in the venom gland of centipede venom glands |
| 8 | Hypothesis | This was a discovery project - with no pre-conceived idea of toxin distribution |
| 9 | Abstract | doi: 10.1073/pnas.1424068112 |
| 10 | Data | Completely Available |
| 11 | Data Negative | No |
| 12 | Data Link | In the process of making the data set available |
| 13 | Data Format | Bruker Daltonics, imzML |
| 14 | Data Files | 28052013-SNG-slide-1-2-4X; 28052013-SNG-slide-1-2-4X.imzML; 28052013-SNG-slide-1-2-4X.ibd |
| 15 | Dataset Names | 28052013-SNG-slide-1-2-4X |
| 16 | Sample Type | Centipede venom gland |
| 17 | Sample List | 28052013-SNG-slide-1-2-4X |
| 18 | Sample Disambiguation | NA |
| 19 | Surface Type | ITO slide |
| 20 | Surface Mods | NA |
| 21 | Clinical | NA |
| 22 | Species | *Scolopendra morsitans* |
| 23 | Age | Adult |
| 24 | Gender | NA |
| 25 | Bio Sampling | Manual dissection |
| 26 | Post Mortem | 0 |
| 27 | Stabilization Type | Fixation (RCL2) |
| 28 | Storage | Samples stored in paraffin blocks |
| 29 | Orientation | Longitudinal |
| 30 | Embedding | Paraffin |
| 31 | Microtome | Microtome |
| 32 | Mounting | Melted |
| 33 | Thickness | 7 |
| 34 | Wash | Paraffin removed by xylene wash |
| 35 | Visualization | H&E |
| 36 | Scan | Yes |
| 37 | Scan Res | NA |
| 38 | Scan Instrument | Olympus microscope with camera (DP26) |
| 39 | Scan Type | Same |
| 40 | *InSitu* Chem Type | NA |
| 41 | *InSitu* Instrument | NA |
| 42 | *InSitu* App | NA |
| 43 | *InSitu* Reagent | NA |
| 44 | *InSitu* Solution | NA |
| 45 | MS Model | ultraFlex III |
| 46 | MS Vendor | Bruker Daltonics |
| 47 | Ionization | MALDI |
| 48 | Polarity | Positive |
| 49 | MS Customization | NA |
| 50 | Voltages | IS1 25 kV, IS2 23.2 kV, Lens 6.5, Deflection suppressing up to 980 Da |
| 51 | Extraction | Yes, 100 ns |
| 52 | Analyzer | ToF |
| 53 | Reflectron | No - linear |
| 54 | Detector | Linear, electron multiplier |
| 55 | Control Software | flexControl |
| 56 | Control Version | 3.3 |
| 57 | Min Range | 1000 |
| 58 | Max Range | 9000 |
| 59 | Cal Type | External |
| 60 | Cal Standard | Clinprot mixture (Bruker) |
| 61 | Cal Min | 1046 |
| 62 | Cal Max | 12600 |
| 63 | Shots | 200 |
| 64 | Speed | NA |
| 65 | Matrix | alpha-cyano-4-hydroxycinnamic acid (CHCA) |
| 66 | Matrix Instrument | ImagePrep |
| 67 | Matrix App | Spray |
| 68 | Matrix Amount | 7 mg/mL |
| 69 | Matrix Solvent | 50% ACN, 0.2% TFA |
| 70 | Laser Type | Smartbeam 1 (355nm) |
| 71 | Laser Diameter | 50 |
| 72 | Laser Repetition | 0.2 (200 Hz) |
| 73 | Align | flexImaging co-registration |
| 74 | Spatial Res | 50 |
| 75 | Oversampling | No |
| 76 | Scan Type | Horizontal line scan |
| 77 | Scan Pattern | Zigzag |
| 78 | Scan Direction | Left to right, then right to left |
| 79 | Scan Line Direction | NA |
| 80 | Delocalization | No - determined by whether analyte was detected off tissue or not |
| 81 | Process Software | ClinProTools; SCiLS Lab |
| 82 | Process Version | 3.0 ; 2015b |
| 83 | Processing | Normalization, Peak Alignment, pLSA |
| 84 | Noise Estimation | TopHat Baseline Subtract |
| 85 | Baseline Sub | Yes |
| 86 | Baseline Sub Type | TopHat Baseline Subtract |
| 87 | Smooth | Yes |
| 88 | Smooth Type | Gauss |
| 89 | Smooth Width | 2 |
| 90 | Smooth Other | 4 cycles |
| 91 | Normalization | RMS |
| 92 | Peak Picking | No |
| 93 | Peak Picking Type | NA |
| 94 | SNR | NA |
| 95 | Peak Picking Other | NA |
| 96 | Analysis Software | flexImaging, SCiLS Lab |
| 97 | Analysis Version | 4.1; 2015b |
| 98 | MSI Peak Eval | NA |
| 99 | MSI Bin Size | 1 *m/z* |
| 100 | MSI Measured Theo | Measured |
| 101 | MSI Interpolation | No |
| 102 | Quant Method | NA |
| 103 | Quant Reagents | NA |
| 104 | Quant Procedure | NA |
| 105 | ID Mz | Yes |
| 106 | ID MSMS | No |
| 107 | ID LCMSMS | Proteomics, Transcriptomics |
| 108 | ID Mass Tol | Approx. 2 Da |
| 109 | Other | NA |

**Table S4.** Application of first generation MIAMSIE to data-set report.

| *MIAMSIE ID no.* | *Field name* | *Data-set* |
| --- | --- | --- |
| 1 | Researcher | Michael Becker |
| 2 | Organizational Unit | A DMPK, G DM&T, L DM&T6 |
| 3 | Institution | Boehringer Ingelheim Pharma GmbH & Co KG, Biberach a.d. Riss, GERMANY |
| 4 | Ethics Approval | NA |
| 5 | Contact | Michael_[4.becker@boehringer-ingelheim.com](mailto:4.becker@boehringer-ingelheim.com) |
| 6 | Title | MALDI MSI analysis of BI X and its metabolite in rat eyes |
| 7 | Aim | Investigate suitability of embedding medium |
| 8 | Hypothesis | NA |
| 9 | Abstract | The primary objective of this analysis is to detect the parent compound BI X and its metabolites and their localization in the investigated tissue, and to evaluate to compatibility of the embedding medium Traganth with MALDI MSI measurements |
| 10 | Data | NA, not available |
| 11 | Data Negative | NA |
| 12 | Data Link | NA |
| 13 | Data Format | NA |
| 14 | Data Files | NA |
| 15 | Dataset Names | Slide 327_l+r_BI-X[100mg]_DHB_Rat_Eye.mis |
| 16 | Sample Type | Rat, Eye |
| 17 | Sample List | 16BXXX_A421_Dosed, 16BXXX_A121_Control |
| 18 | Sample Disambiguation | NA |
| 19 | Surface Type | ITO coated glass slide |
| 20 | Surface Mods | NA |
| 21 | Clinical | Animal necropsy |
| 22 | Species | *Rattus norvegicus* |
| 23 | Age | NA |
| 24 | Gender | Female |
| 25 | Bio Sampling | NA |
| 26 | Post Mortem | NA |
| 27 | Stabilization Type | Freezing in N2(l)-cooled isopentane |
| 28 | Storage | < 12 months at -80°C |
| 29 | Orientation | NA |
| 30 | Embedding | 2% Traganth |
| 31 | Microtome | Thermo Fisher HM 560MV |
| 32 | Mounting | Thaw-mounted |
| 33 | Thickness | 12 |
| 34 | Wash | None |
| 35 | Visualization | Scanning, H&E, virtual microscopy |
| 36 | Scan | Yes |
| 37 | Scan Res | 3200 |
| 38 | Scan Instrument | Reflecta PF scanner |
| 39 | Scan Type | Same |
| 40 | *InSitu* Chem Type | NA |
| 41 | *InSitu* Instrument | NA |
| 42 | *InSitu* App | NA |
| 43 | *InSitu* Reagent | NA |
| 44 | *InSitu* Solution | NA |
| 45 | MS Model | MS Model Bruker solariX XR 7T |
| 46 | MS Vendor | Bruker Daltonik GmbH |
| 47 | Ionization | MALDI |
| 48 | Polarity | Positive |
| 49 | MS Customization | NA |
| 50 | Voltages | NA |
| 51 | Extraction | NA |
| 52 | Analyzer | FTICR |
| 53 | Reflectron | NA |
| 54 | Detector | NA |
| 55 | Control Software | ftms Control |
| 56 | Control Version | 2.2.127 |
| 57 | Min Range | 100 |
| 58 | Max Range | 3000 |
| 59 | Cal Type | External + lock mass |
| 60 | Cal Standard | NaTFA + DHB |
| 61 | Cal Min | 158.9640 |
| 62 | Cal Max | 1246.7624 |
| 63 | Shots | 100 |
| 64 | Speed | NA |
| 65 | Matrix | DHB |
| 66 | Matrix Instrument | HTX Technologies TM-sprayer |
| 67 | Matrix App | Spray |
| 68 | Matrix Amount | 20 mg/ml, 4,8 mg/1700 mm^2^ |
| 69 | Matrix Solvent | 60% methanol with 0.1% TFA |
| 70 | Laser Type | Smartbeam II (355nm) |
| 71 | Laser Diameter | 50 |
| 72 | Laser Repetition | 2000 Hz |
| 73 | Align | flex-Imaging teaching |
| 74 | Spatial Res | 50 |
| 75 | Oversampling | No |
| 76 | Scan Type | Discrete spots, horizontal lines |
| 77 | Scan Pattern | Flyback |
| 78 | Scan Direction | Left to right |
| 79 | Scan Line Direction | Top to bottom |
| 80 | Delocalization | Yes |
| 81 | Process Software | NA |
| 82 | Process Version | NA |
| 83 | Processing | Online data reduction, Peak list generation |
| 84 | Noise Estimation | NA |
| 85 | Baseline Sub | NA |
| 86 | Baseline Sub Type | NA |
| 87 | Smooth | NA |
| 88 | Smooth Type | NA |
| 89 | Smooth Width | NA |
| 90 | Smooth Other | NA |
| 91 | Normalization | RMS |
| 92 | Peak Picking | Yes |
| 93 | Peak Picking Type | NA |
| 94 | SNR | 1 |
| 95 | Peak Picking Other | NA |
| 96 | Analysis Software | flexImaging, SCiLS Lab, Excel |
| 97 | Analysis Version | 5.0.78; 2016b |
| 98 | MSI Peak Eval | Height |
| 99 | MSI Bin Size | 3 mDa |
| 100 | MSI Measured Theo | Highest mass in window based on theoretical |
| 101 | MSI Interpolation | No |
| 102 | Quant Method | No |
| 103 | Quant Reagents | NA |
| 104 | Quant Procedure | NA |
| 105 | ID Mz | Parent and known metabolite |
| 106 | ID MSMS | No |
| 107 | ID LCMSMS | LC MS/MS of tissue homogenate |
| 108 | ID Mass Tol | < 3 mDa |
| 109 | Other | NA |

**Table S5.** MSIcheck standard with the corresponding MIAMSIE ID, Name and Description provided.

| *MIAMSIE ID no.* | *Field name* | *Description* |
| --- | --- | --- |
| 1 | Contact | Stable (ideally *long-term*) contact information for experimenter(s). |
| 7 | Data | Can data be downloaded without restriction from a repository (PRIDE) or viewed on a Cloud platform (SCiLS Cloud, metaspace2020)? |
| 9 | Data Link | URL, link or resource identifier for the experimental data. |
| 13 | Sample Type | The type of sample (animal model organ, clinical, geological). |
| 16 | Surface Type | Surface analyzed or used as an underlying substrate for analysis (ITO, glass, metal) |
| 17 | Surface Mods | Was the surface analyzed modified in any way (functionalized, coated)? |
| 21 | Ionization | Ionization type used (MALDI, LDI, SALDI, SIMS, LAESI, DESI). |
| 22 | Polarity | Polarity (positive, negative). |
| 26 | Analyzer | Mass analyzer used (ToF, Q-ToF, LTQ, Ion Trap, Orbitrap, FTICR). |
| 31 | Control Software | Name of MS control software. |
| 32 | Control Version | Version of MS control software. |
| 33 | Min Range | Minimum *m/z* for the acquired mass range. |
| 34 | Max Range | Maximum *m/z* for the acquired mass range. |
| 36 | Cal Type | Type of calibration employed (external, internal, lock mass). |
| 37 | Cal Standard | Calibration standard or standard mixture(s) used. |
| 55 | Stabilization | What stabilization method was used (dehydration, heat inactivation, alcohol/cross-linking fixation, freezing)? |
| 57 | Embedding | Embedding method for sample (OCT, CMC, gelatin, paraffin, none). |
| 62 | Microtome | (Cryo)-microtome instrument used to section material. |
| 65 | Thickness | Tissue section thickness (µm) |
| 67 | Wash | Wash or solution treatment for section(s) and/or surface(s) (fixation, re-hydration). |
| 69 | Visualization | The visualization or histological staining method applied to the sample (H&E, scanning, photography). |
| 71 | Scan | Was sample / section scanned prior to analysis for co-registration? |
| 81 | Matrix Type | What compound or material was used to mediate ionization (none, chemical, metal, nanoparticles, other)? |
| 82 | Matrix Instrument | Instrument used for matrix preparation (manual sprayer, ImagePrep, sublimation chamber, iMatrixSpray, TM-sprayer). |
| 88 | Laser Diameter | Laser spot size, or measured area (µm). |
| 94 | Spatial Res | The size of the data pixels (spectra) [µm]. |
| 102 | Process Software | Name of pre-processing software |
| 103 | Process Version | Version of pre-processing software |
| 104 | Processing | List of the pre-processing steps / pipeline used on the raw data. |
| 112 | Normalization | Method used for intensity normalization (none, TIC, RMS). |
| 120 | MSI Bin Size | Mass filter width / range or bin size (*m/z*) for plotted ion images. |
| 127 | ID MSMS | Was MS/MS on tissue performed? Are spectra included? |

**Table S6.** Research leader MSIcheck (*Yes / No / NA / Limited*) of two publications.

| *Field name* | *Research leader (ref #1)* | *Check (ref #1)* | *Research leader (ref #2)* | *Check (ref #2)* |
| --- | --- | --- | --- | --- |
| Contact | Yes | Yes | Yes | Yes |
| Data | No | No | No | No |
| Data Link |  | No | No | No |
| Sample Type | Yes | Yes | Yes | Yes |
| Surface Type | Yes | Yes | Yes | Yes |
| Surface Mods | No | Yes | No | Yes |
| Ionization | Yes | Yes | Yes | Yes |
| Polarity | Yes | No | Yes | Yes |
| Analyzer | Yes | Yes | Yes | Yes |
| Control Software | Yes | Yes | Yes | Yes |
| Control Version | Yes | Yes | Yes | Yes |
| Min Range | Yes | Yes | Yes | Yes |
| Max Range | Yes | Yes | Yes | Yes |
| Cal Type | No | Yes | Yes | Yes |
| Cal Standard | Yes | Yes | Yes | No |
| Stabilization | Yes | Yes | Yes | Yes |
| Embedding | Yes | Yes | Yes | Yes |
| Microtome | Yes | Yes | No | No |
| Thickness | Yes | Yes | Yes | Yes |
| Wash | Yes | Yes | Yes | Yes |
| Visualization | Yes | Yes | Yes | Yes |
| Scan | No | No | No | No |
| Matrix Type | Yes | Yes | Yes | Yes |
| Matrix Instrument | Yes | Yes | Yes | Yes |
| Laser Diameter | No | No | No | No |
| Spatial Res | Yes | Yes | Yes | Yes |
| Process Software | Yes | Yes | Yes | No |
| Process Version | Yes | Yes | Yes | No |
| Processing | Yes | Limited | Yes | Limited |
| Normalization | Yes | Yes | Yes | Yes |
| MSI Bin Size | Yes | No | Yes | Limited |
| ID MSMS | No | No | Yes | Yes |

**Table S7.** Papers found using the PubMed search string *"(("2017/5/1"[Date - Publication] : "2017/6/1"[Date - Publication])) AND maldi AND imaging"* on the 3rd of July 2017.

| *Publication* | *Reference list number* | *Inclusion status* |
| --- | --- | --- |
| da Silva Araujo *et al.* | 35 | Included |
| Banno *et al.* | 36 | Included |
| Berry *et al.* | - | Not included – full text not available |
| Bradshaw *et al.* | 37 | Included |
| Brentan Silva *et al.* | 38 | Included |
| Briggs *et al.* | - | Not included – protocol |
| Chen *et al.* | 39 | Included |
| Cho *et al.* | 40 | Included |
| Crecelius *et al.* | 41 | Included |
| Gonzalez de San Roman *et al.* | 42 | Included |
| Eckelmann *et al.* | 43 | Included |
| Ermini *et al.* | 44 | Included |
| Griffiths *et al.* | - | Not included – review |
| Hamilton *et al.* | 45 | Included |
| Hansen and Lee | 46 | Included |
| Harvey | - | Not included – review |
| Karlsson and Hanrieder | - | Not included – review |
| Kim *et al.* | 47 | Included |
| Lasch and Noda | 48 | Included |
| Lee *et al.* | 49 | Included |
| Malys and Owens | - | Not included – inclusion debatable |
| Maronpot *et al.* | - | Not included – opinion piece |
| ORourke *et al.* | 50 | Included |
| Rivas *et al.* | 51 | Included |
| Sandrin and Demirev | - | Not included – review |
| Sui *et al.* | - | Not included – behind a paywall |
| Teklezgi *et al.* | 52 | Included |
| Wu *et al.* | - | Not included – not MSI |
| Wu *et al.* | 53 | Included |
| Xu *et al.* | - | Not included – not MSI |
| Yamamoto *et al.* | - | Not included – not MSI |

**Element S8.** R script required for plotting of MSIcheck review results.

library(plyr)

library(ggplot2)

library(stringr)

v.df = read.csv("./MSIcheck_values_290318_notabs.txt", header=TRUE, sep=",", stringsAsFactors = FALSE)

v.df <- v.df[,1:3]

f.rem <- 'Paper'

v.tmp <- subset(v.df, !(Name %in% f.rem))

v.tmp[is.na(v.tmp$Code), 'Code'] = 'Not Applicable'

v.sum <- ddply(v.tmp, c("Name", "Code"), summarise, Count = length(paper.id))

v.sum$Code <- factor(v.sum$Code, levels = c("No", "Yes", "Limited", "Not Applicable"))

m.tmp = reshape(v.sum, direction = 'wide', idvar = 'Name', timevar = 'Code')

names(m.tmp)[2:5] = substring(names(m.tmp)[2:5], 7)

names(m.tmp)[names(m.tmp) == "Not Applicable"] = "NotA"

m.tmp[is.na(m.tmp)] = 0

o.tmp = m.tmp[order((m.tmp$Yes + m.tmp$Limited)/ (m.tmp$Yes + m.tmp$Limited + m.tmp$No),

m.tmp$NotA, m.tmp$Yes, decreasing = c(TRUE, TRUE, TRUE)), "Name"]

v.sum$Name = factor(v.sum$Name, levels = o.tmp)

p = ggplot(data=v.sum) +

geom_bar(stat="identity", aes(Name, Count, fill=Code)) +

#colour reference: <http://sape.inf.usi.ch/quick-reference/ggplot2/colour>

scale_fill_manual(values=c("red", "steelblue2", "green1", "gray46")) +

xlab("MSIcheck Field Name") +

theme_bw() +

theme(axis.text.x=element_text(size=11,angle=90,hjust=1,vjust=0.5),

axis.text.y=element_text(size=11), axis.title = element_text(size=15),

legend.title = element_text(size=15), legend.text = element_text(size=15))

ggsave(file.path("./", "MSIcheck.png"), p, dpi = 300)
